# The potential of HBV cure: an overview of CRISPR-mediated HBV gene disruption

**DOI:** 10.3389/fgeed.2024.1467449

**Published:** 2024-10-09

**Authors:** Zhi Q. Yao, Madison B. Schank, Juan Zhao, Mohamed El Gazzar, Ling Wang, Yi Zhang, Addison C. Hill, Puja Banik, Jaeden S. Pyburn, Jonathan P. Moorman

**Affiliations:** ^1^ Center of Excellence in Inflammation, Infectious Disease and Immunity, James H. Quillen College of Medicine, East Tennessee State University, Johnson City, TN, United States; ^2^ Department of Internal Medicine, Division of Infectious, Inflammatory and Immunologic Diseases, Quillen College of Medicine, ETSU, Johnson City, TN, United States; ^3^ Hepatitis (HBV/HCV/HIV) Program, James H. Quillen VA Medical Center, Department of Veterans Affairs, Johnson City, TN, United States

**Keywords:** HBV, cccDNA, CRISPR/Cas9, gene editing, gene therapy

## Abstract

Hepatitis B virus (HBV) infection is a common cause of liver disease worldwide. The current antiviral treatment using nucleotide analogues (NAs) can only suppress *de novo* HBV replication but cannot eliminate chronic HBV infection due to the persistence of covalently closed circular (ccc) DNA that sustains viral replication. The CRISPR/Cas9 system is a novel genome-editing tool that enables precise gene disruption and inactivation. With high efficiency and simplicity, the CRISPR/Cas9 system has been utilized in multiple studies to disrupt the HBV genome specifically, eliciting varying anti-HBV effects both *in vitro* and *in vivo*. Additionally, multi-locus gene targeting has shown enhanced antiviral activity, paving the way for combination therapy to disrupt and inactivate HBV cccDNA as well as integrated HBV DNA. Despite its promising antiviral effects, this technology faces several challenges that need to be overcome before its clinical application, i.e., off-target effects and *in vivo* drug delivery. As such, there is a need for improvement in CRISPR/Cas9 efficiency, specificity, versatility, and delivery. Here, we critically review the recent literature describing the tools employed in designing guide RNAs (gRNAs) targeting HBV genomes, the vehicles used for expressing and delivering CRISPR/Cas9 components, the models used for evaluating CRISPR-mediated HBV gene disruption, the methods used for assessing antiviral and off-target effects induced by CRISPR/Cas9-mediated HBV gene disruption, and the prospects of future directions and challenges in leveraging this HBV gene-editing approach, to advance the HBV treatment toward a clinical cure.

## Introduction

Hepatitis B virus (HBV) is a hepatotropic, non-cytopathic DNA virus that has chronically infected more than 300 million people worldwide, with approximately 1.5 million new infections per year (World Health Organization, 2024; Schweitzer et al., 2015; Ott et al., 2017). HBV is a common cause of liver disease, including chronic hepatitis, liver cirrhosis, and hepatocellular carcinoma (HCC), resulting in one million deaths annually (Peng et al., 2012; Arbuthnot and Kew, 2001). Despite universal HBV vaccinations and the availability of antiviral therapy, chronic HBV infection remains a disturbing public health problem, causing life-threatening diseases globally. In this review, we summarize the literature describing the molecular biology of chronic HBV infection and current treatments, followed by a discussion of HBV gene editing technology as a new strategy to disrupt the HBV genome. The objective of this review is to foster the development and application of this novel gene editing technology for HBV gene therapy to advance the treatments toward clinical HBV cure.

### HBV cccDNA and antiviral treatment

Current antiviral treatments using pegylated interferon (Peg-IFN) or nucleot(s)ide analogues (NAs) can only suppress *de novo* HBV replication but cannot eliminate chronic HBV infection. In addition, these treatments are hampered by the emergence of viral mutations and thus drug resistance. This inability of the treatments to completely clear HBV is primarily driven by the persistence of the covalently closed circular (ccc) DNA that sustains HBV replication (Gish et al., 2015; Allweiss and Dandri, 2017; Nassal, 2015). In the absence of complete viral eradication, HBV rebound and hepatitis flares will invariably occur following treatment interruption or cessation.

Distinct from integrated HBV DNA that may only encode subgenomic transcripts (e.g., HBsAg), cccDNA has a minichromosomal structure that is extremely stable within infected hepatocytes (Wang et al., 2022; Wang et al., 2020). It possesses a unique ability to self-amplify via the intracellular nuclear re-import machinery (known as intracellular amplification) (Xia and Guo, 2020; Ko et al., 2018), serving as a template for transcription of all HBV mRNAs, including the pre-genomic RNA (pgRNA) that is crucial for viral genome replication (Zoulim, 2005; Shi and Zheng, 2020). Thus, HBV cccDNA is the key therapeutic target for eradicating HBV infection. So far, the molecular mechanisms governing HBV cccDNA biogenesis in infected hepatocytes remain elusive, and there are no available drugs that can directly target HBV cccDNA (Guo and Guo, 2015; Lucifora and Protzer, 2016; Zhu et al., 2019; Dandri and Petersen, 2020). Therefore, novel curative strategies (e.g., genetic approaches), focusing on disruption and inactivation of the HBV cccDNA as well as integrated HBV DNA, are urgently needed to eliminate chronic HBV infection (Kwon and Lok, 2011; Liang et al., 2015; Allweiss et al., 2023; Fanning et al., 2019; Dusheiko, 2020).

### HBV genome and life cycle

**FIGURE 1:**
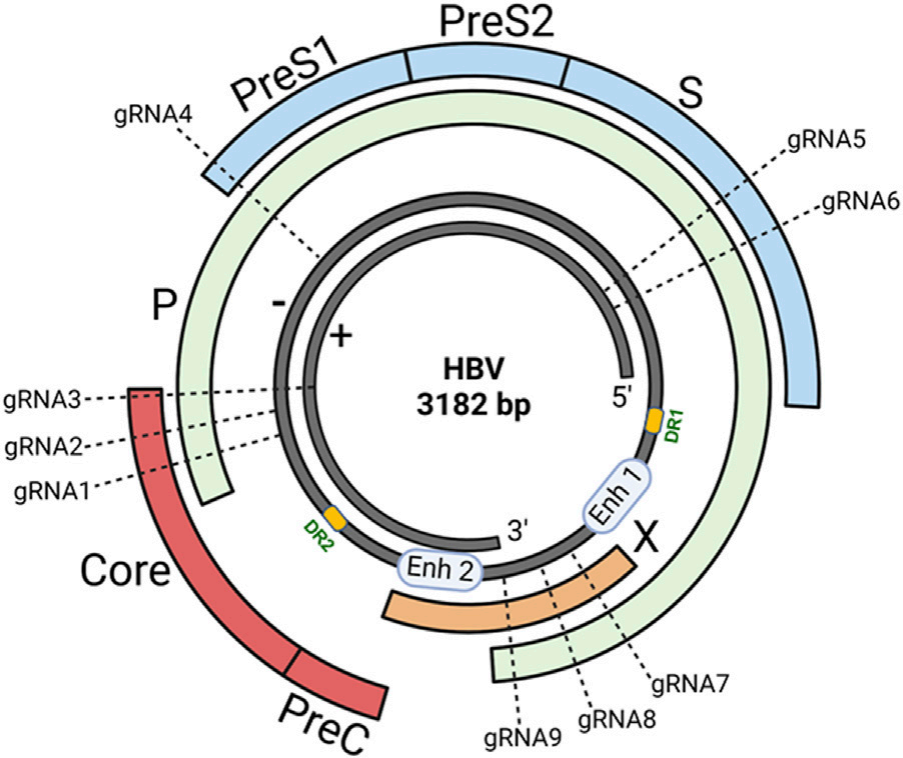
HBV genome and gRNAs design. The genome of HBV is a double-stranded (+/−) circular DNA of about 3.2 kb pairs comprising four overlapping open reading frames (ORFs): surface (S), capsid (C), polymerase (P), and X genes. To select the most specific targets in the HBV genome, we used an online CHOPCHOP program to analyze HBV sequences that have the best-predicted on-target and lowest off-target effects. We compared the genomic DNA sequences of different HBV genotypes originating from distinct geographic regions worldwide and identified 9 target sites in highly conserved regions of the HBV genome (based on the ayw strain, Genbank accession number: NC_003977.2) within the *S*, *C*, *P*, and *X* genes. The sequence alignments of the gRNA target sites on the HBV genome are shown in our recent publication (Suzuki et al., 2021).

HBV, a member of the *Hepadnaviridae* family, is a small DNA virus with unusual features similar to retroviruses (Gish et al., 2015; Allweiss and Dandri, 2017; Nassal, 2015). The genome of HBV is a double-stranded circular DNA of approximately 3.2 kb in length, comprised of an incomplete non-coding positive strand (i.e., relaxed circular DNA/rcDNA) and a complete coding negative strand, with four overlapping open reading frames (ORFs) of surface (S), capsid (C), polymerase (P), and X genes (Figure 1). The *S* gene encodes the hepatitis B surface antigen (HBsAg) that can be structurally and functionally divided into the pre-S1, pre-S2, and S proteins. The *C* gene encodes either the hepatitis B core antigen (HBcAg) or hepatitis B e antigen (HBeAg), depending on whether translation is initiated from the core or pre-core regions, respectively. The *P* gene encodes viral polymerase (pol) with three domains: the terminal protein domain, the reverse transcriptase (RT) domain, and the ribonuclease H domain. The *X* gene encodes hepatitis B x antigen (HBxAg) with multiple functions, including signal transduction, transcriptional activation, and oncogenic ability. Other functionally important elements within the HBV genome include two direct repeats (DR1 and DR2) and two enhancers (Enh1 and Enh2), conferring liver-specific viral replication and expression (Gish et al., 2015; Allweiss and Dandri, 2017; Nassal, 2015) (Figure 1).

**FIGURE 2:**
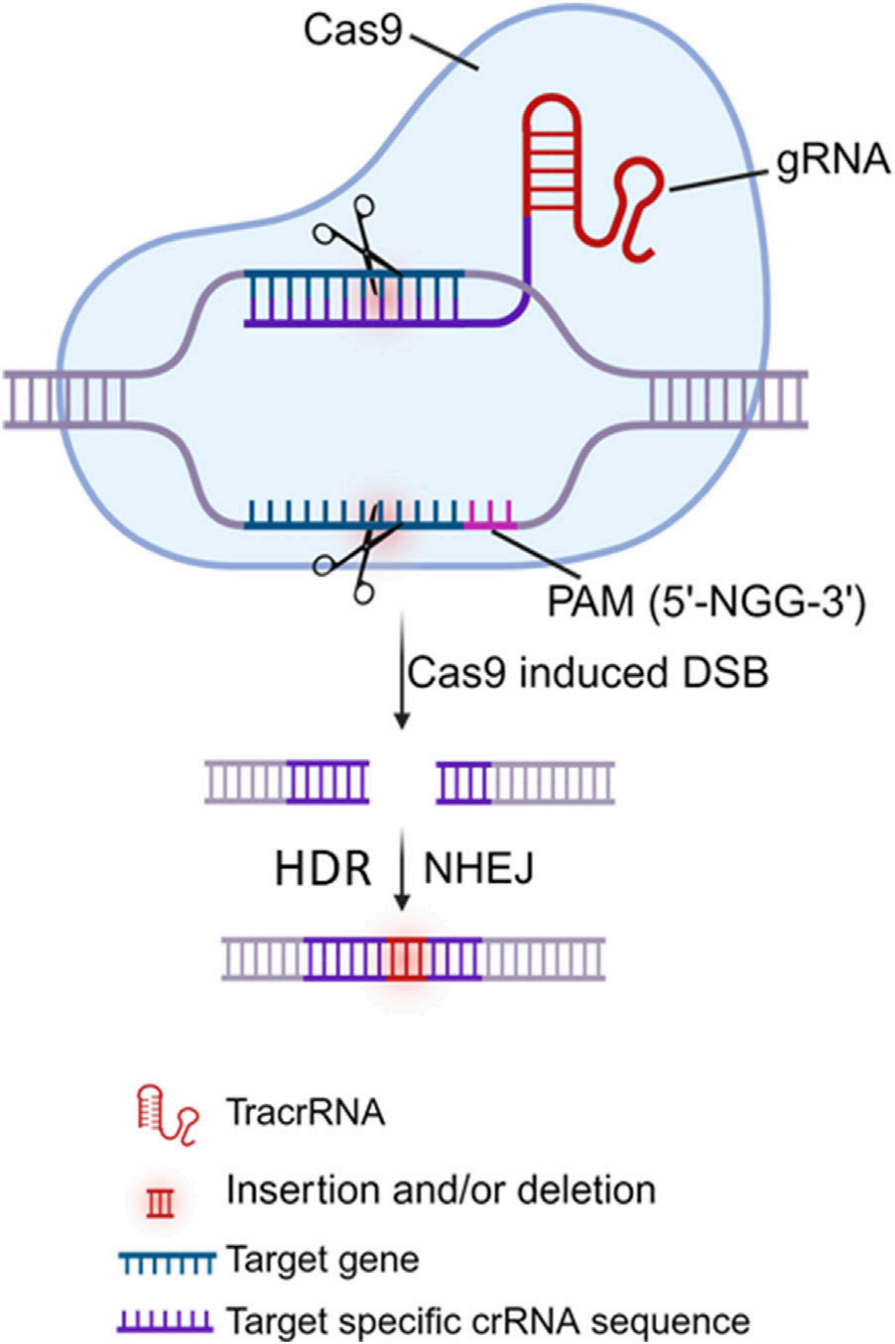
HBV replication and life-cycle in hepatocytes. Upon infection of hepatocytes, the HBV rcDNA is released into the nucleus and converted into cccDNA, which serves as the template for the transcription of viral pgRNA and mRNAs, including X, S, and C RNAs. The RNA transcripts are transported into the cytoplasm and translated into viral proteins. Subsequently, the pgRNA is encapsidated by viral core and polymerase protein, and reverse transcribed into new viral rcDNA. The DNA-containing virus core particles are either enveloped and secreted as progeny viruses or recycled back to the nucleus to amplify the cccDNA pool (i.e., intracellular amplification). A small portion (5%–10%) of HBV DNA can be linearized and integrated into the host genome during this process, forming a replication-incompetent form that can express viral subunit proteins, such as HBsAg.

Upon infection of hepatocytes, viral rcDNA is released into the nucleus and converted into cccDNA, which serves as a template for transcription of viral pgRNA and protein-coding mRNAs, including pre-core RNA, S RNA, and X RNA (Wang et al., 2022; Wang et al., 2020). The viral RNA transcripts are transported into the cytoplasm and translated into viral proteins. Subsequently, pgRNA is encapsulated by viral core and polymerase proteins, and reverse transcribed into new viral rcDNA. The DNA-containing nucleocapsid core particles are either enveloped by S proteins and secreted as progeny virions or recycled back to the nucleus to amplify the cccDNA pool (i.e., intracellular amplification) (Xia and Guo, 2020; Ko et al., 2018) (Figure 2). Notably, a small portion (5%–10%) of HBV DNA can be linearized and integrated into the host genome during this process, forming a replication-incompetent form that can nonetheless express viral subunit proteins (e.g., HBsAg). Because cccDNA sustains HBV chronicity through the continual production of new virions, and integrated HBV DNA contributes to the production of HBsAg (which drives immune evasion and oncogenesis), both cccDNA and integrated HBV DNA are critical targets for HBV gene therapy (Zoulim, 2005; Shi and Zheng, 2020; Guo and Guo, 2015; Lucifora and Protzer, 2016; Zhu et al., 2019; Dandri and Petersen, 2020; Kwon and Lok, 2011; Liang et al., 2015; Allweiss et al., 2023; Fanning et al., 2019; Dusheiko, 2020).

### CRISPR/Cas9-mediated HBV gene editing

Amongst the genetic approaches being employed for targeted gene-editing (Gaj et al., 2013; Cox et al., 2015), including zinc finger nucleases (ZFNs), transcription activator-like effector nucleases (TALENs), and clustered regularly interspaced short palindromic repeats (CRISPR)/CRISPR-associated protein 9 (Cas9) technologies, the CRISPR/Cas9 system is an attractive approach due to its simplicity and design flexibility (Doudna and Charpentier, 2014; Hsu et al., 2014; Jiang and Doudna, 2017; Maepa et al., 2020; Yang and Chen, 2018). Previously, ZFN and TALEN approaches have been utilized to disrupt and inactivate HBV gene replication in HBV cell and animal models, demonstrating that these nucleases could specifically disrupt HBV DNA, reduce pgRNA, and suppress the expression of viral proteins (Cradick et al., 2010; Bloom et al., 2013; Chen et al., 2014). However, targeting viral DNA sequences using ZFN and TALEN technologies requires customization of the DNA-binding domains, which is a complicated and time-consuming process. In contrast, the Cas9 nuclease can be conveniently directed to the target gene by simply designing a guide RNA (gRNA) sequence complementary to the target DNA sequence. Thus, the CRISPR/Cas9 system provides a simple and flexible tool to specifically disrupt and inactivate target genes.

**FIGURE 3:**
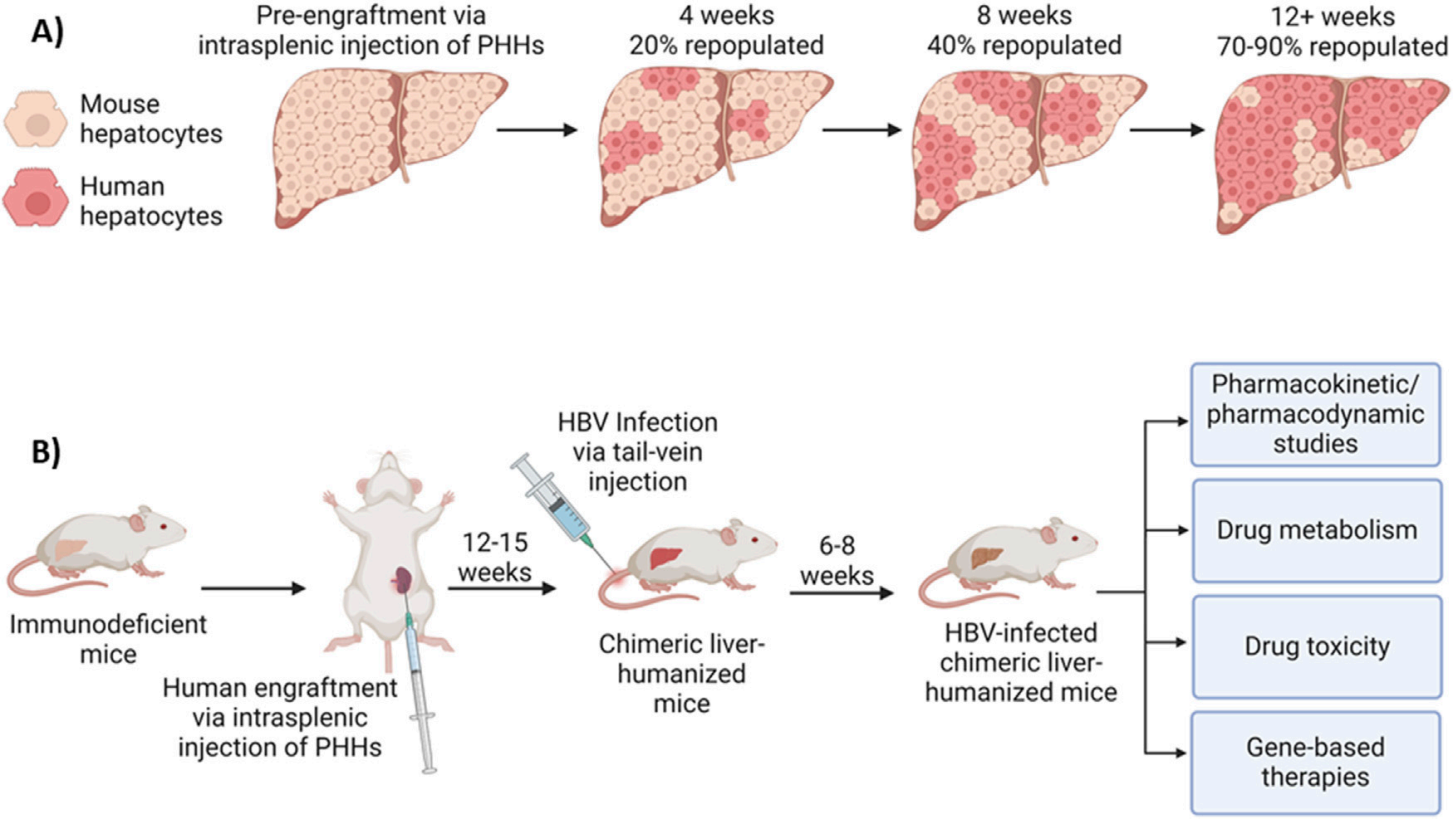
Model of the CRISPR/Cas9 system. The CRISPR/Cas9 gene editing system requires 2 components: the Cas9 nuclease (shown in blue) and the guide RNA (gRNA), comprising of trans-activating CRISPR RNA (tracrRNA) and CRISPR RNA (crRNA) that is complementary to target gene adjacent to the protospacer adjacent motif (PAM, 5′-NGG-3′ where N is non-G). Cas9 complexes with dual gRNA via binding to tracrRNA, whereas crRNA recognition of the target DNA sequence adjacent to the nearby PAM directs Cas9 to induce a double-strand brake (DSB), which is most commonly repaired through the non-homologous end joining (NHEJ) pathway. As error-prone NHEJ repair is not homology-dependent, insertions or deletions (indels) often occur, resulting in disruption and inactivation of the targeted gene.

The CRISPR/Cas9 system comprises two primary components: a gRNA (an RNA sequence of ∼20 nucleotides that can specifically direct/bind to the selected/targeted DNA) and a Cas9 protein (a nuclease that is directed by the gRNA to edit/cleave the target DNA) (Doudna and Charpentier, 2014; Hsu et al., 2014; Jiang and Doudna, 2017). Mechanistically, the Cas9 protein cleaves the target DNA in a sequence-specific manner with the guidance of dual CRISPR RNA (crRNA) and trans-activating CRISPR RNA (tracrRNA). The crRNA-tracrRNA complex can be also fused into a single gRNA (sgRNA) for the Cas9 protein. Target DNA recognition/scission strictly requires i) the presence of a short protospacer adjacent motif (PAM) adjacent to the target site, ii) subsequent R-loop formation and strand scission driven by complementary base pairing between gRNA and target DNA, and iii) Cas9-DNA interactions that lead to double-stranded breaks (DSBs) (Figure 3). The PAM is a specific sequence typically 3 to 8 bp long (depending on which bacterial species the Cas9 proteins are derived from, e.g., *Streptococcus pyogenes*/SpCas9, *Staphylococcus aureus/*SaCas9 or *thermophilus*/StCas9, *Neisseria meningitidis*/NmCas9, and *Francisella novicida*/FnCas9), and PAM is used as a recognition site by Cas9 to initially bind to the target DNA. Theoretically, Cas9 can be directed to specifically cleave any desired genome sequence simply by designing a gRNA that matches the particular sequence of the genome with a downstream PAM (e.g., 5′-NGG-3′ is the most commonly used SpCas9). Once delivered into the cells, the Cas9 nuclease protein can be directed by the gRNA to the target DNA, precisely breaking the double-stranded DNA adjacent to the PAM, resulting in insertion and/or deletion (indel) mutations and even frameshift rearrangement following DNA damage repair, primarily by the error-prone non-homologous end joining (NHEJ) pathway and/or by the accurate but slower homology-directed repair (HDR) pathway (Doudna and Charpentier, 2014; Hsu et al., 2014; Jiang and Doudna, 2017) (Figure 3). Given its specificity, simplicity, and efficiency in gene editing, the CRISPR/Cas9 approach has been employed by many investigators (including us) to target HBV cccDNA for HBV genome destruction and inactivation (Seeger and Sohn, 2014; Lin et al., 2014; Dong et al., 2015; Liu et al., 2015; Ramanan et al., 2015; Zhen et al., 2015; Kennedy et al., 2015; Wang et al., 2015; Karimova et al., 2015; Sakuma et al., 2016; Zhu et al., 2016; Li et al., 2016; Seeger and Sohn, 2016; Li H. et al., 2017; Scott et al., 2017; Jiang et al., 2017; Li H. et al., 2018; Liu et al., 2018; Schiwon et al., 2018; Kostyushev et al., 2019a; Kostyushev et al., 2019b; Kostyusheva et al., 2019; Yang et al., 2020; Kayesh et al., 2020; Murai et al., 2022; Martinez et al., 2022; Suzuki et al., 2021; Zhang et al., 2023). These studies utilized either *in vitro* and/or *in vivo* HBV models and demonstrated varying antiviral effects of the CRISPR/Cas9 system. The use of different gRNA designing tools targeting different HBV genomes, different CRISPR/Cas9 expression and delivery systems, and different methods to detect different HBV products in different HBV cell and animal models, resulted in varying antiviral effects in these studies, which are summarized in Table 1.

**TABLE 1:** Summary of studies utilizing the CRISPR/Cas9 system for HBV disruption and inactivation.

Expression vector	Target regions	HBV models	Evaluation methods	Efficiency	Off target and toxicity	References
Lentivirus	ENII-CP/X, Pre-C, X, X/P	HepG2-NTCP/Cas9, HepAD38, HepG2.2.15	HBcAg immunofluorescence (IF), cccDNA PCR-based sequencing, Sanger sequencing *versus* NGS	8 to 10-fold in HBcAg reduction, >90% HBV DNA was cleaved, indel mutations in PCR products	IFN-induced +, Cas9-induced cytotoxicity	Seeger and Sohn (2014), Seeger and Sohn (2016)
Plasmids	Pre-S1/2, S, P, P, XCp, PCE	Huh-7/HBV plasmid, HDI-HBV model	HBsAg ELISA, HBsAg/HBcAg IF, Plasmid express few cccDNA, 25.6%, 27.8% indels by T7E1	77%–96% HBsAg suppression, 25.6%, 27.8% indels by T7E1	No cytotoxicity, *in vitro* and *in vivo*	Lin et al. (2014)
Plasmids, pX330	X/P, X, C, C/P	Huh-7, HepG2.2.15, cccDNA mouse model	HBsAg/HBeAg ELISA and WB, cccDNA by Southern blot and PCR	60% HBsAg suppression rate, 60%–75% cccDNA reduction	Not evaluated, *in vitro* and *in vivo*	Dong et al. (2015)
Plasmids, pX330	Pre-S1/2, S/P, X/P, P, C, C/P	HepG2/HBV plasmid, HDI-HBV model	HBsAg/HBeAg ELISA, HBV DNA Southern blot and qPCR, 11% mutagenesis by T7E1	11% mutagenesis by T7E1, 100-fold HBV DNA inhibition	Cell viability by CCK8 kit	Liu et al. (2015)
Plasmids, Lentivirus	S, C, X, P	HepG2, Hep-NTCP, HDI-HBV NRG mice	HBV s, e, c Ags by ELISA and IF, pgRNA by PCR, cccDNA by SB, 77%–95% decrease in HBV DNA	>60% HBsAg suppression rate, 77%–95% decrease in HBV DNA	Surveyor assay and deep sequencing	Ramanan et al. (2015)
Plasmids	S, S/P	HBV cell culture, HBV mouse model	HBsAg ELISA and IF, HBV DNA by qPCR, HBV DNA suppression/mutations	HBsAg reduced *in vitro* and *in vivo*, HBV DNA suppression/mutations	Cell viability, not evaluated	Zhen et al. (2015)
Lentivirus	S, C, RT/YMDD	HepAD38, HepaRG	HBsAg ELISA, HBV DNA by real time qPCR, 90% cccDNA reduction, 99% HBV DNA inhibition	90% cccDNA reduction, 99% HBV DNA inhibition	No cytotoxicity, by Promega kit	Kennedy et al. (2015)
Plasmids	Pre-S2, S, P, Pre-C, C, X	Huh7/HBV1.3 plasmid, HepAD38	HBsAg/HBeAg ELISA, PCR-DNA sequencing	>80% HBsAg/HBeAg reduction, HBV (ccc)DNA was cleaved	No cytotoxicity, by MTT	Wang et al. (2015)
Lentivirus	S/P, X/P	HBV reporter plasmid, HepG2.2.15, Hep-NTCP	RFP/GFP fluorescence, HBsAg ELISA/T7E1 assay	Successfully target HBV-sequences, 40%–90% indels/HBV inactivation	Cytotoxicity, not assessed	Karimova et al. (2015)
All-in-one vector/Cas9n	S, X, C	HepG2/HBV plasmid	HBsAg/HBeAg ELISA, Miseq deep sequencing	HBV antigens were suppressed, HBV DNA was cleaved	No off-target mutations	Sakuma et al. (2016)
Plasmids, pX330	S, X	HepG2Huh7/HBV1.3, M-Tg HBV mice	HBsAg ELISA, HBcAg IF/WB, HBV DNA by SB and qPCR	50% HBsAg/HBeAg reduction, HBV DNA suppressed	Cytotoxicity, not assessed	Zhu et al. (2016)
Plasmid pX459, integrated HBV, AAV	S, S/P, X/P, C/P	HepG2.A64 cell line, HDI-/Tg-HBV mice	HBsAg/HBeAg ELISA, HBsAg IF, HBV DNA by qPCR and sequencing	99.9% HBsAg reduction, HBV (ccc)DNA suppressed	Cell viability OK, by CCK-8	Li et al. (2016), Li et al. (2017a), Li et al. (2018a)
ssAAVs	S/P	HepG2-NTCP, SaCas9, HepG2.2.15	HBsAg ELISA, HBV mRNA ddPCR, T7E1 assay, NGS	50%–95%% HBsAg reduction, 46%–61% indels, cccDNA inhibition	No unintended sequence change	Scott et al. (2017)
Cas9 mRNA/gRNA-LLNs	S/P, X/P, pre-C	HepGAD38, HDI-HBV mice	HBsAg/HBeAg ELISA, T7E1 assay, DNA sequencing	Significant HBsAg/HBeAg reduction, HBV mRNA and cccDNA inhibition	No indel, off-target effect	Jiang et al. (2017)
AAV8, SaCas9	S/P, X/P, Pre-C, C/P	HepG2.2.15, HepAD38, HDI-HBV mice	HBsAg/HBeAg ELISA, Deep DNA sequencing	>80% HBsAg/HBeAg reduction, HBV pgRNA and cccDNA inhibition	Undetectable mutations	Liu et al. (2018)
HCAdV, multiplex	P (RT), XCp, RNase H	HepG2.2.15, HepG2-NTCP	HBsAg/HBeAg ELISA, T7E1, RNA/DNA qPCR	54%–76% HBsAg/HBeAg reduction, 39%–78% HBV DNA inhibition	No mutations	Schiwon et al. (2018)
Lentiviral, SpCas9/StCas9	pre-C/C, Enh1, X	HepG2-1.1merHBV, HepG2-1.5merHBV	HBV DNA and cccDNA, HBV pgRNA qPCR	90% HBV cccDNA reduction, Significant HBV pgRNA inhibition, Suppressed HBsAg, HBcAg	Cell viability OK	Kostyushev et al. (2019a), Kostyushev et al. (2019b), Kostyusheva et al. (2019)
BE lentiviruses	S, P, C, X	HBV-HEK293T cells, HepG2.2.15, HepG2-NTCP	Sanger and MiSeq sequencing, HBsAg ELISA, qPCR	>50% base-editing (BE) efficacy,60% HBV DNA inhibition	No genome mutation	Yang et al. (2020)
AAV2 vector	S, P, C, X	HepG2.2.15, HepG2-NTCP, humanized chimeric mice	HBsAg ELISA, qPCR	Suppressed HBsAg, HBcAg, HBV DNA/cccDNA *in vivo*	No cytotoxicity	Kayesh et al. (2020)
lentiviral vector	S, C, X, P	HepG2-NTCP-C4-iCas9, PHHs	whole genome sequencing, HBV DNA/RNA qPCR, NGS	2-log HBV cccDNA reduction, 50% cccDNA inhibition in PHHs	Cell viability, Not assessed	Murai et al. (2022)
gRNA/Cas9, RNPs	S, C, X, P	HepG2-NTCP, PHHs	HBsAg/HBeAG ELISA, qRT-PCR, SB, DNA/RNA sequencing	Indel formation, generate episomal variants	Cell viability, Not assessed	Martinez et al. (2022)
AAV2 and LNP-based Cas9, Cas12 RNPs Synthetic gRNA, synthetic Cas9 RNPs	Pre-C S, C, X, P	HepG2-NTCP-30 HepG2.2.15, HepDE19, HepG2-NTCP	HBV DNA and cccDNA HBsAG/HBeAG ELISA, HBV (ccc)DNA qPCR	60%–80% inhibition of HBV DNA and cccDNA LNP-RNPs are more efficient than AAV2 approach 50% HBsAg reduction, 95% cccDNA inhibition	No cytotoxicity No cytotoxicity	Suzuki et al. (2021) Zhang et al. (2023)

In the following sections, we discuss the tools used to design gRNAs targeting HBV genomes, the vehicles used for expression and delivery of CRISPR/Cas9 components, the models used for evaluation of CRISPR-mediated HBV gene-editing, the methods used for assessing antiviral and off-target effects of CRISPR-mediated HBV gene-editing, and the prospects for future directions and challenges in using this gene editing approach for HBV gene disruption and inactivation.

## Tools used for designing gRNAs targeting HBV genomes

Identification of gRNAs for unique Cas9 nucleases to cleave the HBV genome without causing off-target effects is the initial and critical step in designing CRISPR/Cas9-based therapeutics for HBV gene disruption and inactivation. A high rate of viral replication and lack of proofreading during reverse transcription result in a high potential for HBV to mutate and develop quasispecies (Croagh et al., 2015). Due to the high heterogeneity of the HBV genome, HBV is categorized into different genotypes and sub-genotypes with sequence divergence of 4% to >8%, respectively (Croagh et al., 2015). The heterogeneity of the HBV genome (i.e., HBV quasispecies) complicates the design of gRNAs needed to target multiple or all HBV genotypes and subtypes in viral gene disruption.

### Designing gRNAs targeting HBV conserved regions

HBV is classified as a para-retrovirus and is likely to mutate at an estimated rate of 1–3 × 10^-5^ nucleotide substitutions per site per year during reverse transcription (Chan, 2011), leading to a high complexity of quasispecies within individual patients (Li et al., 2015). Thus, the most common strategy for disrupting HBV genome by the CRISPR/Cas9 approach involves designing gRNAs with sequences complementary to the conserved regions of the HBV genomes, targeting various genotypes and strains originating from different geographic regions worldwide (Croagh et al., 2015). Using ClustalW multiple alignment in the BioEdit program (Hall, 1999), the alignment analysis of whole genomes of 10 HBV genotypes (A-J) and subtypes (e.g., ayw) (Table 2) can reveal several consensus sequences along the viral genomes. Theoretically, targeting highly conserved regions within the HBV genome increases the likelihood of successful cleavage across different HBV genotypes and reduces the impact of viral mutations, and targeting overlapping regions (e.g., S overlapping P gene, C overlapping P gene, or X overlapping P gene) can enhance efficiency, as cleavage in one region might disrupt multiple viral functions (Ramanan et al., 2015; Karimova et al., 2015; Seeger and Sohn, 2016; Li H. et al., 2017; Zhang et al., 2023). Other regions used for designing gRNAs include gene replication and expression regulatory elements, such as DR1, DR2, Enh1, Enh2, core promoter (critical for transcription of the pre-core and core genes), and the tyrosine-methionine-aspartate-aspartate (YMDD) motif (critical for the activity of the polymerase) (Seeger and Sohn, 2014; Kennedy et al., 2015; Kostyushev et al., 2019b; Kostyusheva et al., 2019; Yang et al., 2020). Because gRNAs are designed to target the conserved regions of the HBV genome, it is reasonable to speculate that the CRISPR/Cas9 system that inhibits the replication of a specific HBV genotype (e.g., genotype D, subtype ayw) may also target other genotypes and subtypes. For example, Liu et al. (2015) designed gRNAs targeting the relatively conserved regions of different HBV genotypes listed in the world organization reference panel and demonstrated that one gRNA could target the viral replication of different HBV genotypes (e.g., A, B, C, D) and sub-genotypes. Kennedy et al. (2015) reported that targeting the HBV RT domain was particularly effective in HBV inactivation. They reported that lentiviral vectors encoding combinations of gRNAs designed to target the HBV RT, S, and C genes effectively suppress viral cccDNA accumulation in chronically HBV-infected cells and inhibit *de novo* HBV infection. Moreover, these gRNAs showed an additive inhibitory effect on HBV DNA accumulation when used in combination with known pharmacological RT inhibitors (Kennedy et al., 2015). A combination of CRISPR-mediated gene disruption and NA (entecavir)-mediated RT suppression to enhance the overall antiviral effect has also been reported by Kayesh et al. (2020). Therefore, this strategy could avoid missing the targets of the CRISPR/Cas9 system caused by mutations during HBV replication because the conserved regions imply a lower variability within that region of the viral template. This strategy also increases the inhibitory efficiency of the CRISPR/Cas9 system due to the importance of the conserved regions after natural selection during viral evolution.

**TABLE 2:** Hepatitis B virus (HBV) genotype and subtype.

Accession number	Genotype	Genome Length (bp)	Definition
HE974362.1	HBV genotype A1	3,221	HBV genotype A1 complete genome, isolate Mart-B01
HE974364.1	HBV genotype A2	3,221	HBV genotype A2 complete genome, isolate Mart-B15
AB981583.1	HBV genotype B	3,215	HBV genotype B complete genome, isolate P2-121214
LC456132.1	HBV genotype C	3,215	HBV genotype C complete genome, isolate CAM-HB31
HE815465.1	HBV genotype D	3,182	HBV genotype D, serotype ayw3, complete genome
HE974384.1	HBV genotype E	3,212	HBV genotype E complete genome, isolate Mart-B84
DQ823095.1	HBV genotype F	3,215	HBV genotype F complete genome, isolate BA45
AB625342.1	HBV genotype G	3,248	HBV genotype G complete genome, isolate MEX918M
AB275308.1	HBV genotype H	3,215	HBV genotype H complete genome
AF241411.1	HBV genotype I	3,215	HBV genotype I complete genome, isolate 8290
AB486012.1	HBV genotype J	3,182	HBV genotype J complete genome, isolate JRB34
NC003977.2	HBV subtype ayw	3,182	HBV subtype (genotype D) ayw complete genome

### Designing gRNAs using computational tools

Another strategy for designing gRNAs with predicted ranking of specificity and potency is to use computational tools. The online tools commonly used for designing CRISPR gRNAs are summarized in Table 3. These computational tools are designed to analyze HBV sequences with the best-predicted on-target effects (i.e., HBV sequences critical for viral replication/expression) and lowest off-target effects (i.e., minimum homology with the human genome) for different Cas9 nucleases. While HBV-infected hepatocytes contain various forms of viral DNA, cccDNA is the key target for disrupting/eradicating HBV replication/infection. Because cccDNA is converted from rcDNA with removal of the 5′-end of minus and plus strands of the whole HBV DNA sequence, this shorter cccDNA sequence complicates the search for a PAM gene. Thus, it is more practical to design gRNAs using the whole HBV genome as a template to increase the chance of CRISPR/Cas9-mediated gene disruption and inactivation. The gRNAs designed using this strategy can target both the integrated HBV DNA and cccDNA. Nevertheless, the accessibility of Cas9 protein to cccDNA is another factor for consideration in gRNA design (Ramanan et al., 2015).

**TABLE 3:** Web-based tools for gRNA design.

Tool name	Website	Purpose	Features
CHOPCHOP	https://chopchop.cbu.uib.no/	User-friendly tool for designing gRNAs across multiple CRISPR systems	Analyzes on-target efficacy, off-target predictions, and secondary structures in the gRNA
CRISPOR	http://crispor.gi.ucsc.edu/	Widely used tool for detailed gRNA design and off-target prediction	Generates gRNA candidates, predicts off-target sites, and provides specificity scores and structural visualization
E-CRISP	http://www.e-crisp.org	Interface for designing gRNAs for various organisms with detailed scoring	Provides on-target efficacy and off-target potential analyses
CRISPR-ERA	http://crispr-era.stanford.edu/	Design gRNAs for various CRISPR systems with comprehensive analyses	Identifies target sites, assesses off-target effects, and scores gRNA efficacy and specificity
Cas-Designer	http://www.rgenome.net/cas-designer/	Robust platform supporting multiple CRISPR systems	Evaluates on-target activity and off-target potential comprehensively
CRISPRdirect	https://crispr.dbcls.jp/	Simple tool for designing gRNAs with minimal off-target effects	Identifies target sites quickly with a user-friendly interface
CCTop	http://crispr.cos.uni-heidelberg.de	Design CRISPR/Cas9 gRNAs with high specificity and minimal off-target effects	Identifies target sites, predicts off-target effects, scores gRNA efficiency with an easy-to-use interface
GuideScan	https://guidescan.com/	Focuses on maximizing on-target activity and minimizing off-target effects	Identifies and scores potential gRNAs with thorough off-target prediction

Given the advantages of the CRISPR/Cas9 system by its convenience for implementation of multiplex gRNAs to target multiple DNA sites simultaneously, several studies have shown that using a combination of gRNAs could substantially enhance the overall antiviral effect (Lin et al., 2014; Dong et al., 2015; Liu et al., 2015; Ramanan et al., 2015; Kennedy et al., 2015; Wang et al., 2015; Karimova et al., 2015; Sakuma et al., 2016; Liu et al., 2018; Zhang et al., 2023). In addition, the application of multiplex gRNA/Cas9 treatment may mitigate viral evasion (which can occur with single gRNA/Cas9 treatment), thereby increasing anti-HBV effectiveness.

In our lab, we utilized the web-based program (CHOPCHOP (Labun et al., 2019)) to select the most specific target sites for disrupting the HBV genome and compared the genomic DNA sequences of different HBV genotypes (Croagh et al., 2015) from GenBank originating from distinct geographic regions worldwide. We identified 9 target sites in highly conserved regions of the HBV genome within the P, C, S, and X genes (Figure 1). We synthesized 9 gRNAs (20-nt long, based on the ayw strain; Genbank accession number: NC_003977.2) tailored to target HBV genome adjacent to the PAM (5′-NGG-3′), which is recognized by SpCas9 with a nuclear localization signal (NLS) that can direct the synthetic gRNA/Cas9 ribonucleoprotein (RNP) into the cell nucleus. We tested the antiviral activities and off-target effects of these synthetic gRNAs/Cas9 RNPs in different HBV cell models (HepG2.2.15, HepDE19, and HepG2-NTCP) and identified the most potent and specific gRNAs that can significantly suppress HBV replication without eliciting discernible cytotoxicity in the treated hepatocytes (Zhang et al., 2023). We are currently investigating the *in vivo* antiviral and adverse effects of these selected/candidate gRNAs using HBV-infected animal models.

## Vehicles used for expression and delivery of CRISPR/Cas9 components

While CRISPR/Cas9 is the most promising approach for disruption/inactivation of target genes, it requires therapeutic administration of the two components needed for the expression and delivery of both gRNA and Cas9 components into the same (target) cell. Both viral and non-viral vectors had been utilized for the expression and delivery of CRISPR/Cas9 components in HBV-infected hepatocytes (Seeger and Sohn, 2014; Lin et al., 2014; Dong et al., 2015; Liu et al., 2015; Ramanan et al., 2015; Zhen et al., 2015; Kennedy et al., 2015; Wang et al., 2015; Karimova et al., 2015; Sakuma et al., 2016; Zhu et al., 2016; Li et al., 2016; Seeger and Sohn, 2016; Li H. et al., 2017; Scott et al., 2017; Jiang et al., 2017; Li H. et al., 2018; Liu et al., 2018; Schiwon et al., 2018; Kostyushev et al., 2019a; Kostyushev et al., 2019b; Kostyusheva et al., 2019; Yang et al., 2020; Kayesh et al., 2020; Murai et al., 2022; Martinez et al., 2022). Recently, synthetic gRNA/Cas9 and gRNA/Cas12 ribonucleoproteins (RNPs) have also been used for HBV gene disruption and inactivation (Suzuki et al., 2021; Zhang et al., 2023).

### Non-viral vectors

In early studies, gRNA and Cas9 were expressed and delivered into human hepatoma cell lines (such as Huh7 or HepG2) or mouse models (BALB/c or C57/B6) by plasmid vectors via cell transfection *in vitro* and hydrodynamic injection (HDI) through tail vein *in vivo* (Lin et al., 2014; Dong et al., 2015; Liu et al., 2015; Ramanan et al., 2015; Zhen et al., 2015; Wang et al., 2015; Zhu et al., 2016; Li et al., 2016). The most commonly used plasmid vectors for expressing CRISPR/Cas9 components are summarized in Table 4. Using plasmids for expression and delivery of CRISPR/Cas9 components is expedient and convenient both *in vitro* and *in vivo*. Alternatively, CRISPR/Cas9 modalities can be delivered into hepatocytes via non-viral vectors such as lipid nanoparticles. For instance, due to the long persistence and immunogenicity of plasmid and viral vectors in the host that prevent their wide application in humans, Jiang et al., 2017 used lipid-like nanoparticles (LNPs) to deliver gRNA and Cas9 mRNA to mice receiving HBV-expression plasmids by HDI and showed decreases in HBsAg, HBeAg, and HBV DNA levels. This is the first report of non-viral LNP delivery of CRISPR/Cas9 components (gRNA plus Cas9 mRNA) in animals. Recently, Suzuki et al. (2021) also used LNPs loaded with ribonucleoprotein-oligonucleotide complexes and demonstrated robust genome editing and HBV inhibition. The optimized formulation significantly suppressed both HBV DNA and cccDNA in HBV-infected human hepatocytes. Therefore, LNP-based CRISPR delivery represents a significant contribution to the development of CRISPR technology and its practical applications in gene editing therapy.

**TABLE 4:** The most commonly used plasmid vectors for expression of CRISPR/Cas9 targeting HBV genome.

Name	Features	Promoter	Selection marker	Applications
pX330-U6-Chimeric BB-CBh-hSpCas9	Single plasmid expressing both Cas9 and gRNA	U6 (gRNA), CBh (Cas9)	None intrinsic	Targeting HBV DNA for gene disruption and functional inactivation
pX458 (pSpCas9 BB-2A-GFP)	Cas9 and gRNA expression with GFP reporter	U6 (gRNA), CBh (Cas9)	GFP	Gene disruption of HBV DNA, monitoring transfection efficiency in HBV-infected cells
pX459 (pSpCas9 BB-2A-Puro)	Cas9 and gRNA expression with puromycin resistance	U6 (gRNA), CBh (Cas9)	Puromycin resistance	Gene disruption of HBV DNA, creation of stable cell lines resistant to HBV
pX601 (AAV-CMV: SaCas9-U6)	Smaller SaCas9 for AAV delivery	U6 (gRNA), CMV (SaCas9)	None intrinsic	*In vivo* editing of HBV DNA using AAV delivery systems
lentiCRISPR v2	Lentiviral vector for stable integration and expression	U6 (gRNA), EF1α or CMV (Cas9)	Puromycin resistance	Long-term expression in HBV-infected cells, *in vivo* HBV models
pCW-Cas9	Doxycycline-inducible Cas9 expression	U6 (gRNA), Tet (Cas9)	None intrinsic	Controlled activation of Cas9 nuclease in HBV gene editing, reducing off-target effects
pUC19-based gRNA and Cas9 Cloning Vectors	Simple vectors for cloning/expressing gRNAs and Cas9	U6 (gRNA)	None intrinsic	Custom gRNA design targeting specific HBV sequences
pXPR_003 and pLX311-Cas9 (sgRNA/Cas9 Cloning Vectors)	High-throughput cloning vectors for gRNAs and Cas9	U6 (gRNA)	None intrinsic	High-throughput screening of gRNAs targeting HBV

### Viral vectors

Besides plasmid vectors, viral vectors are also used for the expression and delivery of CRISPR components in HBV cell and animal models (Seeger and Sohn, 2014; Kennedy et al., 2015; Seeger and Sohn, 2016; Scott et al., 2017; Liu et al., 2018; Schiwon et al., 2018; Kostyushev et al., 2019a; Kostyushev et al., 2019b; Kostyusheva et al., 2019; Yang et al., 2020; Kayesh et al., 2020; Murai et al., 2022; Martinez et al., 2022). Adeno-associated virus (AAV) vectors are the ideal tools for expressing and delivering CRISPR components that can modify target genes in a broad range of host cell and tissue types (Bijlani et al., 2022). AAV is a replication-defective, non-pathogenic virus that can infect both dividing and non-dividing cells with high efficiency and is safe for use in humans (Flotte, 2004; Bak and Porteus, 2017). AAV vectors are promising vehicles for expression and delivery of CRISPR/Cas9 modalities for HBV gene therapy because of their non-pathogenicity, non-integrating nature, potential for being produced at very high titers (up to 10^14^ virion particles/mL), and for their specific AAV serotype (2, 3, and 8) with high degree of liver tropism as well as high infection rate in hepatocytes. However, AAV packaging capacity is constrained (approximately 4.6 kb), limiting its versatility in packaging CRISPR/Cas9 cassettes given that the SpCas9 gene from *S. pyogenes* alone is approximately 4.3 kb (not including gRNA and AAV regulatory cassettes) (Bak and Porteus, 2017; Mout et al., 2017). SaCas9, StCas9, and NmCas9 are all shorter than SpCas9 by > 1 kb and thus can be easily packaged into the AAV vector, but their antiviral activities remain to be tested in HBV animal models, including the liver-humanized HBV infection mouse model. Scott et al. (2017) and Liu et al. (2018) reported using AAVs containing cassettes encoding SaCas9 and gRNAs targeting the HBV genome and demonstrated successful inactivation of cccDNA and inhibition of HBsAg production without unintended sequence mutations in HBV-infected hNTCP-HepG2 cells and HBV-HDI mice. Alternatively, high-capacity adenoviral vectors (HCAdVs, which can package up to 36 kb of foreign genes) devoid of all coding genes are powerful tools to deliver large DNA cargos into cells. Schiwon et al. (2018) reported using HCAdVs to deliver multiplexed (three) CRISPR/Cas9 targeting cassettes against the HBV genome and demonstrated a significant reduction in HBV antigen and cccDNA levels. However, AAV vectors are limited by their high immunogenicity, potentially inducing strong immune responses to both viral vectors and Cas9 protein.

In addition to AAV vectors, lentiviruses are also employed for the expression and delivery of CRISPR/Cas9 components. For example, Seeger C. et al. (Seeger and Sohn, 2014; Seeger and Sohn, 2016) utilized a lentivirus vector to establish a HepG2-NTCP cell line that is permissive to HBV infection. To combine the HBV infection system with the CRISPR/Cas9 platform for viral gene editing, they introduced Cas9 into HBV-infected (via HepAD38 supernatant) HepG2-NTCP cells with a second lentivirus vector where Cas9 expression is regulated by the CMV-tetracycline (tet) promoter that is inducible with doxycycline. To evaluate the potential antiviral activity of CRISPR/Cas9 against HBV, they infected HepG2-NTCP/Cas9 cells with a third lentivirus expressing individual gRNAs targeting HBV. The HBV-infected cells were maintained in a culture medium containing doxycycline to induce Cas9 expression, followed by an examination of HBV gene disruption and inactivation through PCR-based cccDNA sequencing and IF-based HBcAg reduction/suppression. Similar to AAVs, lentivirus-based expression systems are advantageous in that they have a high rate of transfection and are able to infect both dividing and nondividing cells. However, the potential integration of lentiviral components into the host genome can induce insertional mutagenesis and unwanted off-target effects. Additionally, these viral delivery approaches can induce immunogenicity due to sustained Cas9 expression and antiviral immunity. Thus, although lentivirus vectors possess the ability to mediate potent transduction and stable expression both *in vitro* and *in vivo* in laboratory settings, biosafety, ethical, and public health concerns limit their application in humans.

Despite the challenges associated with packaging large gene editor-encoding sequences into viral vectors, recent advances in the field are overcoming these obstacles. The most common approach for solving this problem is to split the therapeutic genes into two AAV packages. Alternatively, smaller Cas9 variants (such as SaCas9, StCas9, and NmCas9) may be used if a single package is desired. HCAdVs present an additional option. Consequently, the translating viral vector-mediated gene editing against HBV from the bench to clinical application is feasible and reachable. Further insights into delivering HBV targeting designer nucleases using viral vectors are provided in a recent review article (Jacobs et al., 2022).

Besides expression vectors, delivering CRISPR/Cas9 components into target cells is crucial for its applications. The most commonly used strategies for *in vitro* delivery include transfection of CRISPR/Cas9 expression vectors using chemical reagents such as lipofectamine or polyethylenimine (PEI), electroporation or nucleofection using electric equipment, microinjection, virus-mediated delivery, and using nanoparticles such as LNPs and gold nanoparticles (AuNPs). The most commonly used strategies for *in vivo* delivery of these expression vectors include virus-mediated delivery, nanoparticles, hydrodynamic injection or direct injection, and using *in vivo* electroporation or gene gun. The features and applications of these *in vitro* and *in vivo* methods are summarized in Table 5.

**TABLE 5:** Summary of commonly used delivery methods for CRISPR/Cas9-mediated HBV gene editing.

Category	Methods	Features	Applications
*In vitro* delivery	Chemical Transfection	Chemical compounds facilitate the uptake of CRISPR/Cas9 complexes by cells	Simple and cost-effective for HBV gene editing in cell lines, with lower efficiency compared to other methods
	Electroporation	Brief electric pulses create transient pores in the cell membrane, allowing CRISPR/Cas9 entry	High transfection efficiency for difficult-to-transfect cells, suitable for HBV gene editing in primary hepatocytes and other challenging cell types
	Microinjection	CRISPR/Cas9 components are directly injected into individual cells using a fine needle	Precise delivery method suitable for HBV gene editing in primary cells and single-cell manipulation studies, although labor-intensive and technically challenging
	Virus-mediated delivery (AAV, Lentivirus)	Viral vectors deliver CRISPR/Cas9 genes into cells or target tissues for stable integration or expression	Suitable for long-term HBV gene editing studies, creation of stable HBV knockout cell lines
	Nanoparticles	Nanoparticles encapsulate CRISPR/Cas9 mRNAs, facilitating their uptake by cells	Efficient delivery of CRISPR/Cas9 for HBV gene editing in a wide range of cell types, suitable for high-throughput screening and functional studies
*In vivo* delivery	Virus-mediated delivery (AAV, Lentivirus)	Viral vectors are efficient carriers for delivering CRISPR/Cas9 components to target tissues	*In vivo* delivery of CRISPR/Cas9 for HBV gene editing in animal models, offering stable integration and long-term expression
	Nanoparticles	Encapsulate CRISPR/Cas9 components for efficient delivery to target tissues	*In vivo* delivery of CRISPR/Cas9 for HBV gene editing in liver tissues of animal models, with potential for clinical translation due to biocompatibility
	Hydrodynamic Injection	Rapid injection of a large volume of DNA solution induces transient transfection in the liver	Delivery of CRISPR/Cas9 for HBV gene editing in animal models, suitable for transient expression and rapid screening of gene editing efficacy
	*in vivo* electroporation	Brief electric pulses facilitate the uptake of CRISPR/Cas9 complexes by tissues	*In vivo* delivery of CRISPR/Cas9 for HBV gene editing in liver tissues of animal models, offering efficient transfection without viral vectors
	Microinjection	Direct injection of CRISPR/Cas9 components into target tissues or embryos	Precise delivery of CRISPR/Cas9 for HBV gene editing in animal models, suitable for studies requiring single-cell manipulation or spatial control

The following issues need to be considered in CRISPR/Cas9 delivery: 1) Efficiency: The chosen method should maximize the uptake and expression of CRISPR/Cas9 components in target cells or tissues; 2) Specificity: Targeting specific tissues or cell types to minimize off-target effects and systemic exposure; 3) Safety: Minimizing potential cytotoxicity, immunogenicity, and off-target genome editing; 4) Duration: Depending on the application, transient or stable expression of CRISPR/Cas9 components may be required. Combining these delivery methods with appropriate targeting strategies (e.g., tissue-specific promoters, targeting ligands) can enhance the efficiency and specificity of CRISPR/Cas9 gene editing in both *in vitro* and *in vivo* experimental settings.

### Synthetic gRNA/Cas9 RNPs

The efficient expression and delivery of therapeutic transgenes remain a challenge in gene therapy. Traditional CRISPR/Cas9 expression and delivery systems against HBV often rely on viral or non-viral vectors (Seeger and Sohn, 2014; Lin et al., 2014; Dong et al., 2015; Liu et al., 2015; Ramanan et al., 2015; Zhen et al., 2015; Kennedy et al., 2015; Wang et al., 2015; Karimova et al., 2015; Sakuma et al., 2016; Zhu et al., 2016; Li et al., 2016; Seeger and Sohn, 2016; Li H. et al., 2017; Scott et al., 2017; Jiang et al., 2017; Li H. et al., 2018; Liu et al., 2018; Schiwon et al., 2018; Kostyushev et al., 2019a; Kostyushev et al., 2019b; Kostyusheva et al., 2019; Yang et al., 2020; Kayesh et al., 2020; Murai et al., 2022), raising concerns due to long-term or sustained transgene expression (up to 1–2 years *in vivo*), which can lead to unwanted off-target effects, cytotoxicity, immunological responses, indel mutagenesis, or oncogenesis, and thus raising safety concerns for their application in humans. However, synthetic gRNA/Cas9 RNPs offer an alternative non-viral transient formulation with multiple advantages, including rapid DNA cleavage, decreased off-target effects, low risk of indel mutagenesis, easy synthesis and gRNA multiplexing, and readiness for clinical use (Roth et al., 2018; Hultquist et al., 2019; Liu et al., 2021). Recently, we and others employed this transient CRISPR/Cas9 approach and demonstrated that synthetic gRNA and Cas9 RNPs are sufficient to disrupt and inactivate HBV cccDNA and reduce the risk of off-target cleavage (Suzuki et al., 2021; Zhang et al., 2023), highlighting their potential for clinical use. Different from long-term expression systems, this transient administration formula may require repeated treatment based on RNP half-life *in vivo*, which should be determined in pre-clinical and clinical pharmacodynamic studies. Existing non-viral vectors that can deliver these gRNA/Cas9 RNPs (e.g., liposomes or LNPs) face several obstacles, such as off-site delivery, limited cargo-loading capacity, poor biocompatibility/stability, cytotoxicity, and potential immunogenicity (Jiang et al., 2017; Suzuki et al., 2021; Nguyen et al., 2020; Wessel et al., 2019; Chen et al., 2019; Wang et al., 2017). These challenges limit the *in vivo* use of synthetic gRNA/Cas9 RNPs for clinical applications. Thus, designing specific gRNA/Cas9 RNP modalities and developing novel vehicles for their delivery are urgently needed for their applications in eradicating chronic HBV infection (Zhang et al., 2021; Nelson and Gersbach, 2016; Li L. et al., 2018).

Exosomes are a subtype of nanoscale membranous vesicles naturally released from the endocytic compartment of live cells, and their cargos (DNA, RNA, proteins, and lipids) are reflective of their cell-of-origin (Kalluri and LeBleu, 2020). Exosomes are considered promising delivery vehicles for gRNA/Cas9 RNP (Lin et al., 2018; Ye et al., 2020) (given the large size of RNPs) because they circumvent most of the limitations associated with the currently available viral and non-viral vectors. For example, immune responses to Cas9 protein and viral vectors are a major concern in CRISPR/Cas9-mediated gene therapy, as they can compromise the effectiveness of the drug or potentially cause serious side effects (Charlesworth et al., 2019; Simhadri et al., 2018; Wagner et al., 2019). Distinct from viral vectors, exosome-mediated delivery of synthetic gRNA/Cas9 RNPs robustly overcomes immunogenicity concerns as it provides immune-privileged protection. Compared to other non-viral delivery systems such as synthetic nanoparticles, exosomes are immunologically inert and non-cytotoxic, if purified from a compatible cell source (Mendt et al., 2018). Unlike liposomes, exosomes boast various membrane-anchored proteins that can prolong their circulation half-life by evading phagocytic clearance, facilitating efficient cellular uptake and cargo delivery to the recipient cell (Kamerkar et al., 2017). Notably, exosomes can cross stringent biological barriers and can be engineered to deliver encapsulated gRNA/Cas9 RNPs specifically to target cells. Previous studies have demonstrated the ability of engineered exosomes to deliver therapeutics for targeted cancer therapy (Kim et al., 2017; Zhang et al., 2022). Thus, engineered exosomes may serve as an ideal vehicle for the delivery of synthetic gRNA/Cas9 RNPs specifically to HBV target cells to achieve HBV (ccc) DNA excision, degradation, and inactivation. We have engineered exosomes carrying a small peptide targeting the HBV receptor - sodium taurocholate co-transporting polypeptide (NTCP) - expressed on human hepatocytes. The ability of our engineered exosomes to package synthetic gRNA/Cas9 RNP and deliver it to HBV target hepatocytes is currently under investigation in our lab. One potential advantage of synthetic medicine is that once the specific/potent components are determined, these gRNA/Cas9 therapeutics can be synthesized in large quantities and packaged in hepatocyte-derived exosomes (which can be produced in large quantities using the FiberCell system) for patient treatment.

## Models used for evaluation of CRISPR-mediated HBV gene editing

Researchers have been investigating the application of CRISPR/Cas9 to target/disrupt and functionally inactivate HBV (ccc)DNA for over a decade. Highly representative and relevant HBV cell and animal models are required for the evaluation of CRISPR/Cas9-mediated gene disruption and inactivation. Over the past decade, several HBV cell culture systems have been developed and utilized. These include non-infection models and natural infection models. Non-infection models include HBV plasmid DNA transfection (in which plasmid constructs are introduced into the cells to represent HBV infection but few cccDNA copies are produced) and HBV genome-integrated stable cell lines (in which integrated HBV DNA in stable cell lines is cleaved, such as HepDE19 and HepAD38). Natural infection models, including primary human hepatocytes (PHHs), HepaRG, and HepG2-NTCP cells, allow for true cccDNA formation. Overall, the sensitivity and ease of establisment of CRISPR-mediated HBV gene editing ranks as follows (from highest to lowest): 1) plasmid-transfection models, 2) HBV-integrated models, and 3) natural infection models. However, despite having the lowest sensitivity and highest complexity of CRISPR-mediated HBV gene editing, natural infection models have the closest mimic settings to the CRISPR-mediated gene disruption and inactivation *in vivo*.

### HBV-expression plasmids and reporter genes

Earlier studies only demonstrated the utility of the CRISPR/Cas9 technology in disrupting HBV-expression plasmid (Lin et al., 2014; Dong et al., 2015) or HBV-flanked with reporter genes (RFP or GFP (Karimova et al., 2015)), either in Huh-7 human hepatoma cells co-transfected with an HBV-expression vector (and gRNA/Cas9-expression cassettes) or in a mouse model receiving HBV-expression plasmids by HDI, which is quite different from the circular minichromosomal cccDNA that exists in infected hepatocytes. Though convenient for assessing CRISPR-mediated HBV gene editing, HBV-expression plasmids and/or reporter genes do not faithfully mimic natural HBV infection, with few cccDNA copies generated in the transfected systems (Lin et al., 2014; Wang et al., 2015). Because cccDNA plays an important role in HBV persistence, reactivation after treatment withdrawal, and drug resistance, cccDNA is the major barrier to the eradication of chronic HBV infection. Considering that few cccDNA copies existed in Huh7 cells after transfection with HBV-expression plasmids, Dong et al., 2015 developed a cccDNA model in Huh7 cells, in which cccDNA was generated by co-transfecting the HBV precursor plasmid precccDNA with pCre/LoxP-mediated recombination (Qi et al., 2014). Technically, a loxP-chimeric intron was engineered into a monomeric HBV genome in a precursor plasmid and there was a residual loxP site existing in the HBsAg/L ORF upon Cre recombination. However, the loxP site between the 5′ intron and 3′ intron was spliced during viral transcription, so it is functionally seamless with this HBV cccDNA model (Qi et al., 2014). This recombinant cccDNA approach was designed to tackle a very basic question: how to eradicate HBV cccDNA without real HBV infection.

### HBV stable cell lines

The HepG2.2.15 cell line has been utilized as a model for decades in evaluation of CRISPR-mediated HBV gene editing and antiviral effect (Seeger and Sohn, 2014; Dong et al., 2015; Karimova et al., 2015). This HBV cell line harbors a functional HBV integrated DNA and presents cccDNA as well as other forms of HBV DNA that constitutively produce HBV products, as evidenced by the secretion of HBsAg, HBeAg, and infectious Dane-like paticles (Sells et al., 1987). Compared with HepG2.2.15, the HepG2.A64 cell line exhibits higher production of HBV antigens, virions, and HBV cccDNA and are easier to cultivate and transfect, and thus has been used to evaluate CRISPR/Cas9-mediated HBV gene disruption and inactivation (Li et al., 2016; Li H. et al., 2017). Additionally, HepDE19 and HepAD38 cell lines (with or without stable transfection of Cas9 plasmid) harboring integrated HBV genome are also used for a similar purpose (Seeger and Sohn, 2014; Kennedy et al., 2015; Wang et al., 2015; Zhang et al., 2023). In the HepDE19 and HepAD38 cell lines, transcription initiation of an integrated HBV linear DNA genome is tightly controlled by a Tet-repressed promoter. When cultured with a Tet-free medium, these cells initiate HBV replication by transcribing HBV pgRNA and mRNAs, leading to the synthesis of substantial levels of HBV cccDNA and the release of DNA-containing viral particles into the culture supernatants. Thus, these cell lines are often used as HBV cell models to test suppression of HBV replication and cccDNA synthesis by CRISPR/Cas9 (Seeger and Sohn, 2014; Kennedy et al., 2015; Wang et al., 2015; Zhang et al., 2023). These cell lines are also used to produce HBV infectious particles in the supernatants for nature infection of other HBV permissive cells. However, it is noteworthy that these cell lines (HepG2.2.15 and HepDE19 or HepAD38) lack the NTCP receptor (Xu et al., 2021), and thus are deficient in new HBV infection. Nonetheless, intracellular amplification mechanisms can lead to increases in HBsAg/HBeAg, pgRNA/mRNA, and cccDNA production in hepatocytes after plating in culture (Zhang et al., 2023), making them suitable for studying the antiviral effects of CRISPR/Cas9 against HBV replication. While these cell models have the advantages of expressing high virologic markers and are easy to use, they do not represent natural HBV infection and thus cannot model viral entry and early cccDNA activity. Therefore, a limitation of using these stable HBV cell lines is that they do not accurately mimic the conditions of HBV-infected hepatocytes in the liver.

### HBV infection cell models

While certain gRNA/Cas9 can efficiently suppress HBV infection by directly cleaving cccDNA, other gRNA/Cas9 can only cleave/edit integrated but not episomal cccDNA, which does not suppress HBV *de novo* infection (Ramanan et al., 2015). Because Cas9 is a large multi-domain protein, one hypothesis for this observation is that particular regions of the HBV genome are differentially accessible to Cas9 protein because of the tightly packed physical architecture of cccDNA. This underscores the importance of the careful selection of gRNA target sites that are not only important for viral replication but also accessible to Cas9 protein, to achieve a sustained disruption of cccDNA. Also, equally important is the use of an HBV model with a natural viral infection that can produce authentic cccDNA to investigate CRISPR/Cas9 therapeutics for HBV treatment.

Efforts to develop strategies that can reduce cccDNA have been hampered by the lack of long-term tissue culture systems that are permissive for natural HBV infection. Theoretically, PHHs infected with HBV inoculum offer the closest *in vitro* model to natural infection; however, PHHs are limited in supply, expensive, and cannot be sub-cultured to observe the long-term effects of CRISPR/Cas9-mediated HBV gene disruption and inactivation. Notably, HepaRG cells are permissive for natural HBV infection with live virus (Gripon et al., 2002) and are an HBV cell model alternative to the HepG2.2.15, HepDE19, and HepAD38 systems, which only model the late stage of the HBV lifecycle (Ladner et al., 1997). HepaRG cells with HBV infection can produce authentic cccDNA and thus have been used for assessing CRISPR-mediated HBV cccDNA suppression (Kennedy et al., 2015); however, its complicated time-consuming and labor-intensive procedures have discouraged researchers from using this HBV cell model. Notably, CYPRIO (https://cryrio.fr) is launching HCLPearls and iPearls with differentiated HepaRG or HepaSH cell lines and stem-cell derived hepatocytes following a partnership with Biopredic International. Using a cutting-edge liquid-core encapsulation technology, they have validated the pooled HepatoPearls with encapsulated spheroids containing multiple (up to 10) human donors, to reduce the inter-donor variability. This new 3D liver organoid model is viable up to 45 days with transporter activity maintained in culture, suitable for a wide range of applications, such as hepatoxicity and metabolism studies, CYP450 assays, imaging, and other specific projects. Whether this 3D cell culture model is susceptible and permissive to HBV infection and can be used for assessing the CRISPR-mediated HBV gene editing remains to be evaluated.

Another hepatoma cell line that can mimic natural HBV infection in producing authentic cccDNA is HepG2 cells transfected with NTCP (the HBV receptor) (Yan et al., 2012; Tong and Li, 2014), which could be infected by HBV concentrated from either HepDE19, HepAD38 or HepG2.2.15 culture supernatants (Seeger and Sohn, 2014; Ramanan et al., 2015; Seeger and Sohn, 2016; Zhang et al., 2023) or patient serum/plasma-derived virus (Ramanan et al., 2015). This HBV model reflects a more accurate representation of HBV infection, as the viral products are derived from the expression of the viral genome under natural HBV infection, and replication of the viral genome depends on the newly formed cccDNA. This model opened the possibility of determining whether cccDNA formed from infectious virus at the early stage of infection can be targeted and disrupted by the CRISPR/Cas9 system. Therefore, HBV-infected HepG2-NTCP cells have been widely used in evaluating CRISPR-mediated HBV gene editing and inactivation (Seeger and Sohn, 2014; Ramanan et al., 2015; Karimova et al., 2015; Seeger and Sohn, 2016; Zhang et al., 2023).

Notably, treatment with interferon-alpha (IFN-α) did not exhibit a measurable effect on the antiviral activity of the CRISPR/Cas9 system in HepG2-NTCP cells, suggesting that Cas9 and NHEJ activities are not affected by induction of an innate immune response with the cytokine (Seeger and Sohn, 2014). On the other hand, inhibition of the NHEJ-mediated DNA repair pathway has been shown to enhance the CRISPR-mediated anti-HBV activity, with a 2-log reduction in cccDNA in the HepG2-NTCP-C4-iCas9 model, which is an HBV infection cell model with inducible Cas9 expression (Murai et al., 2022). In this study, they used a clinically available drug to block the poly [adenosine diphosphate ribose] polymerase (PARP) activity to enhance the effect of CRISPR-mediated HBV inactivation and demonstrated that the combination of CRISPR and a PARP inhibitor (Olaparib) may represent a novel therapy for HBV elimination. In another study, the inhibition of both homologous and nonhomologous DNA double-strand break repair pathways with a DNA-PKC inhibitor (NU7026) increased the elimination of HBV cccDNA by CRISPR/Cas9 system (Kostyushev et al., 2019a; Kostyusheva et al., 2019), suggesting that this strategy may potentially be utilized as a therapeutic approach for HBV eradication.

### HBV animal models

In addition to humans, HBV can naturally infect chimpanzees or macaques and tree shrews (*Tupaia belangeri*) and establish persistent infection. While these non-human primates are the closest animal models to human HBV infection, there are restrictions on their use for antiviral drug evaluation. Alternative animal models with infection of hepadnaviruses that are closely related to HBV, such as woodchuck hepatitis B virus (WHV) and duck hepatitis B virus (DHBV), can be used, but these two viral infections cannot accurately mimic the life-cycle and pathogenesis of HBV infection.

In patients with chronic hepatitis B (CHB), up to 50 copies of cccDNA exist in the nucleus of each infected hepatocyte with a half-life of approximately 30–50 days, and some of these cells undergo epigenetic modifications (Levrero et al., 2009). In addition to cccDNA, integrated HBV DNA also needs to be cleaved to reduce the production of HBsAg that can induce immune evasion and oncogenesis during chronic HBV infection. Interestingly, a recent study reported that the CRISPR/Cas9 system could cleave large-genome DNA viruses (adenovirus and herpes simplex virus) with high efficiency (Bi et al., 2014). Additionally, the CRISPR/Cas9 system has also been utilized to disrupt latent integrated HIV provirus (Ebina et al., 2013). We have reported using synthetic gRNA/Cas9 RNPs to disrupt HIV genes incorporated into human T cell and monocytic cell lines (Khanal et al., 2022). These studies suggested that the CRISPR/Cas9 system can effectively target both the integrated and extrachromosomal viral genomes. Nevertheless, it remains unclear whether episomal HBV cccDNA and integrated DNA can be disrupted with high efficiency by the CRISPR/Cas9 system *in vivo*. As such, animal models that harbor the *bona fide* cccDNA as well as integrated HBV DNA are required for validation of the results obtained from *in vitro* studies.

### Tg-HBV mouse model

Historically, the HBV transgenic (Tg) mouse model was generated by engrafting with human hepatocytes and played a pivotal role in decoding the HBV production cycle *in vivo*. However, HBV-Tg mice are naturally immune-tolerant to HBV products, and their high viral markers are not produced by the actual HBV infection. Additionally, the virus particles cannot enter the liver cells due to the lack of HBV receptor (NTCP) on mouse hepatocytes, and liver injury cannot be induced due to the intrinsic immune tolerance in HBV-Tg mice.

### HDI-HBV mouse model

To address these limitations, a nonintegrated HBV mouse model was generated through an HBV plasmid delivered by HDI via the tail vein. For instance, Li et al., 2016 reported a 2-log reduction in the secreted virus in this HDI-HBV mouse model, in which the gene editor and HBV plasmid were co-injected into the tail vein at high pressure to physically force the DNA into hepatocytes. However, this system is more akin to a transfection model and the HBV DNA templates in this model are not cccDNA. While the HDI-HBV mouse model is simple, cost-effective, and immune-competent, one limitation of using this model is that the high viral copy number is not produced from a natural HBV infection and no cccDNA is detectable (Lin et al., 2014). Several studies have examined the antiviral effect of CRISPR/Cas9 *in vivo* using the HBV-Tg mouse model or by HDI of HBV-expression plasmids,however, these animal models could not generate authentic cccDNA *in vivo* (Lin et al., 2014; Dong et al., 2015; Liu et al., 2015; Ramanan et al., 2015; Zhen et al., 2015; Zhu et al., 2016; Li et al., 2016; Jiang et al., 2017).

### AAV-HBV mouse model

Mouse models with viral vector (e.g., AAV)-expressed/delivered HBV have also been employed. These AAV-HBV models exhibited low toxicity and ease of use in immune-competent mice, but the full HBV life cycle was incomplete as cccDNA was not formed from the HBV rcDNA. Depending on the AAV loading doses, both transient and persistent viremia were observed in the immune-competent mice (Li et al., 2020). We and others have found that the AAV-HBV transfection mouse model will inevitably cause persistent HBV infection and injury in the liver by high dose (5 × 10^10^ genome copy per mouse) viral infection. At this point, the NTCP transgenic mouse cannot support a productive HBV infection. Therefore, alternative methods have been explored to enable animals to express HBV cccDNA, including the chimeric liver-humanized mouse models.

### Liver-humanized HBV mouse model

**FIGURE 4:**
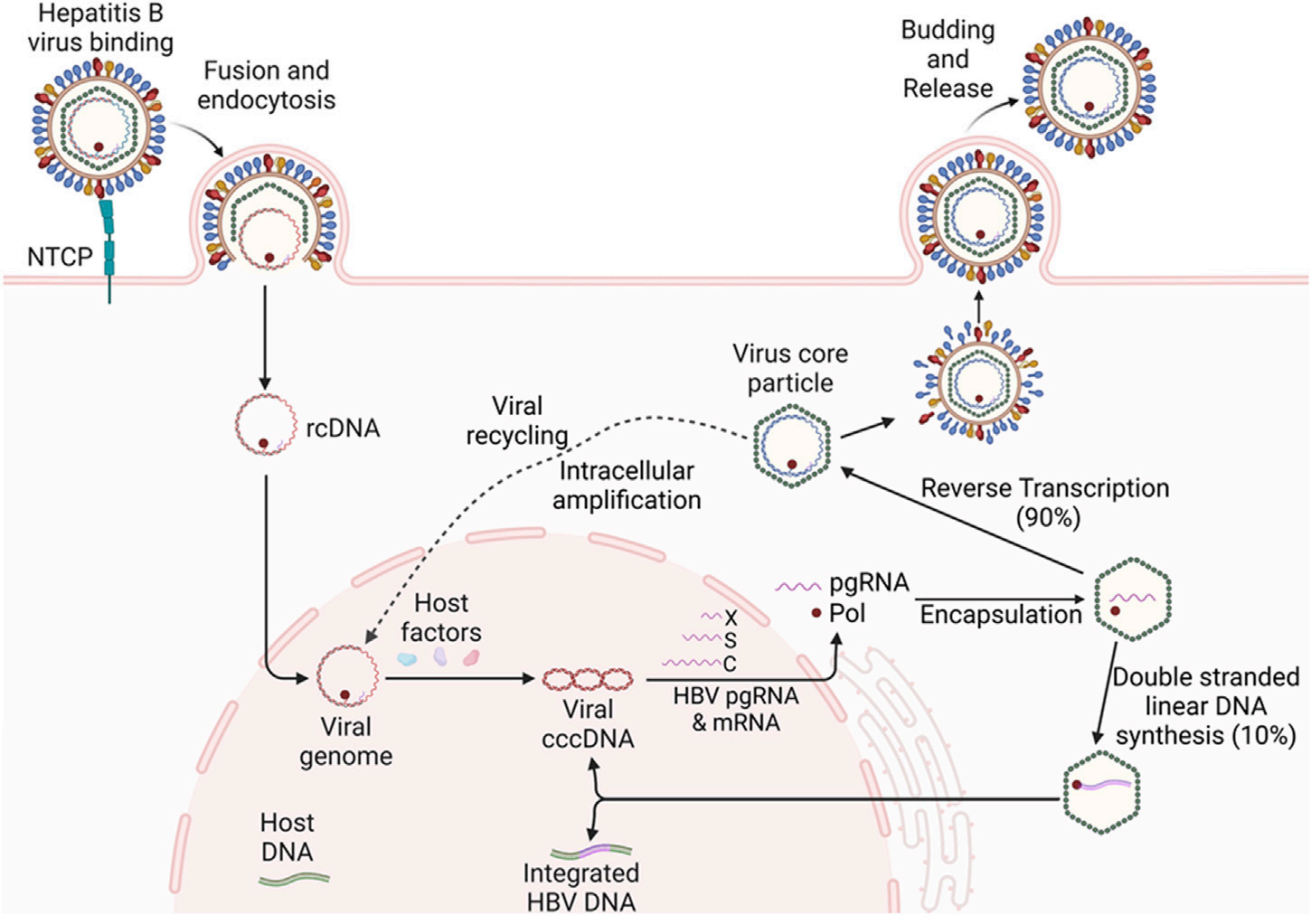
Liver-humanized mouse model for HBV infection and applications. **(A)** Model of mouse liver humanization following engraftment of primary human hepatocytes (PHHs) via intrasplenic injection. Fumarylacetoacetate Hydrolase (FAH) and human albumin immunohistochemistry are often used as markers of hepatocyte repopulation. Humanization levels increase in an exponential manner, with 70%–90% of the liver being humanized within 12–15 weeks. **(B)** Liver-humanized mouse model for HBV infection and applications. Immunodeficient mice are humanized via intrasplenic injection of human hepatocytes, which will then repopulate the host mouse liver with PHHs. After 12–15 weeks, 70%–90% of the mouse liver will be repopulated with human hepatocytes. Following humanization, the mice can be infected with HBV via tail-vein injection. After 6–8 weeks, the mouse can be considered to be persistently infected and used as a model for studying *in vivo* drug transport, antiviral activity/gene-based therapies, pharmacokinetics/pharmacodynamics (PK/PD), drug metabolism, liver toxicity, etc.

HBV fails to naturally infect murine hepatocytes due to blockades at multiple steps of the HBV life cycle (Li et al., 2020). To address the need for a more physiologically relevant model, chimeric liver-humanized mouse models have thus been developed for HBV studies (Li et al., 2020; Sun and Li, 2017; Bissig et al., 2010; Li F. et al., 2017). This type of humanization must be done in immunodeficient mice so that the transplanted human hepatocytes are not rejected. Also, an immunodeficient state and severely damaged endogenous murine hepatocytes create favorable conditions for human hepatocyte proliferation and repopulation. In this case, millions of freshly isolated or cryopreserved and thawed human hepatocytes can be injected into the spleens in these animals, subsequently flowing through the spleen and portal veins into the liver parenchyma, where they ultimately reside and function in the host liver, replacing the endogenous liver with 70%–90% human hepatocytes (Figure 4A). A variety of human hepatic proteins, including fumarylacetoacetate hydrolase (FAH), albumin, apolipoprotein A, and several clotting factors or complements in the plasma can be detected, and their levels are used as biomarkers for the percent of human hepatocyte repopulation in the mouse liver. The resulting humanized liver chimeric mice are susceptible to HBV infection, capable of forming cccDNA, and thus, an excellent model for HBV infection and studying *in vivo* drug transport, antiviral activity, pharmacokinetics/pharmacodynamics (PK/PD) and metabolism, and liver toxicity (Figure 4B).

As an example, the Fah/NRG-hu HEP mice can be generated via engraftment/repopulation with human hepatocytes in NRG-KO (NOD.Rag1KO.IL2RγcKO)/Fah (Fumaryl acetoacetate hydrolase) KO mice (generated by using *in vitro* fertilization and CRISPR/Cas9 technology) and chronically infected with HBV (Bissig et al., 2010), suggesting that this animal model readily supports HBV replication and can be used for testing HBV gene-editing drugs *in vivo*. The immune and liver dual-humanized mice, generated by co-transplantation/engraftment of CD34^+^ human hematopoietic stem cells (HMCs) and hepatic progenitor cells (HPCs), possess both a human immune system and human liver cells that support natural HBV infection and full HBV life cycle are a valuable tool for HBV research and are commercially available (from Yecuris or Phoenixbio), but these immune and liver dual humanized mice are extremely expensive, and also, absence of a complete immune system. Given the limited sources of PHHs derived from humans and the difficulty of culturing and growing/passaging these cells *in vitro*, repopulation and growth of PHHs in liver-humanized mice *in vivo* is employed as an alternative approach to produce large quantities of mice-derived human hepatocytes for research. We are currently testing the use of mice-derived human hepatocytes (HepaSH or HepaRG from Biopredic International) in HBV infection and CRISPR-mediated HBV disruption.

## Methods used for assessing antiviral effects of CRISPR-mediated HBV gene editing

Accurate quantification of HBV products (DNA, RNA, and proteins) is critical for assessing the antiviral effects of CRISPR/Cas9 on HBV gene disruption. Measuring HBsAg and/or HBeAg levels in the supernatants of HBV-infected, CRISPR-treated hepatocytes by ELISA (Lin et al., 2014; Murai et al., 2022) or examining intracellular HBsAg and/or HBcAg by immunofluorescence (IF) microscopy (Seeger and Sohn, 2014; Lin et al., 2014; Seeger and Sohn, 2016), along with HBV (ccc)DNA, mRNA and pgRNA by quantitative PCR (Lin et al., 2014; Murai et al., 2022) are valuable metrics for evaluating antiviral effects of CRISPR-mediated HBV gene editing, whether viral gene disruption or inactivation.

### HBV cccDNA quantification

Several studies reported that the CRISPR/Cas9 system can destroy or inactivate HBV cccDNA (Table 1). It remains challenging to accurately measure HBV cccDNA because rcDNA shares identical sequences with cccDNA and is more abundant (>100-fold) in infected hepatocytes. Historically, Southern blot is the standard method used to distinguish between rcDNA and cccDNA (Lin et al., 2014), but this technique is neither sensitive nor quantitative. In addition, southern blotting requires a relatively large amount of DNA, which may not always be available, especially from clinical samples. The entire process is labor-intensive and time-consuming, making it less practical for high-throughput analysis or rapid diagnostics. It has a limited dynamic range compared to techniques like qPCR or droplet digital polymerase chain reaction (ddPCR), which can more accurately measure a broad range of cccDNA levels. In our studies, we used Hirt DNA extraction combined with DNase digestion, followed by real-time PCR to quantify cccDNA (Zhang et al., 2023). Following CRISPR treatment, we extracted the protein-free HBV DNA from whole cell lysates using the Hirt DNA extraction protocol (Marchetti et al., 2022). The Hirt DNA prep is treated by either plasmid-safe DNase or exonucleases to remove the rcDNA, and the remaining cccDNA is cleaned with a DNA clean and concentrator kit and then subjected to qPCR amplification using primers specific to HBV cccDNA (Zhang et al., 2023). If the cccDNA levels are below the qPCR detection limit, HBV cccDNA can be quantified using ddPCR, which is more specific and sensitive compared to qPCR (Hayashi et al., 2022). The results of ddPCR can be analyzed by Bio-Rad’s Quanta software, which allows the counting of the number of positive and negative droplets. Serial dilutions of HBV plasmid can serve as an external quantification standard to accurately differentiate positive and negative clusters and eliminate false positive droplets.

### HBV intermediate measurement

Demonstrating the disruption of new HBV production requires the assessment of other HBV intermediates/transcripts in its replication cycle. HBV mRNA and pgRNA can be measured by real-time qPCR (Zhang et al., 2023). The amount of HBV RNA in the treated and untreated cells can be also determined using RNAscope, with quantitative analysis performed by QuPath bioimage software. We have used these methods to assess HBV RNA levels in HepDE19 cells treated with gRNA/Cas9 RNPs (Zhang et al., 2023). These novel techniques, in conjunction with ELISA, immunostaining, and flow cytometry analysis of HBV antigens and cell phenotypic markers, can facilitate the detection and phenotyping of actively and inactively HBV-infected cells. To further validate gRNA/Cas9 antiviral capability, we have serially diluted the supernatants from HBV-gRNA/Cas9-treated HepDE19 cells to infect HepG2-NTCP cells and then measured HBV cccDNA and transcripts (pgRNA and HBsAg/HBeAg) (Zhang et al., 2023). This approach can determine the extent of the reduction in HBV particles following the CRISPR-mediated HBV gene disruption and inactivation.

### T7E1 mismatch assay

We (Zhang et al., 2023) and others (Martinez et al., 2022) have previously reported that targeting HBV DNA by CRISPR/Cas9 results in DSBs, which are primarily repaired by the NHEJ pathway and generate transcriptionally active episomal variants. Also, CRISPR/Cas9-mediated cleavage of HBV DNA may result in in-frame or out-of-frame mutations in the host genomic DNA. Thus, detecting DNA cleavage and indel mutations following CRISPR/Cas9-mediated DNA disruption and repair is important for confirming its gene-editing effects. The assay for detecting mismatches by T7 endonuclease 1 (T7E1) is frequently employed to detect specific cleavage sites or indel mutations in the target gene (HBV DNA) in the treated cells (Lin et al., 2014; Dong et al., 2015; Liu et al., 2015; Karimova et al., 2015; Scott et al., 2017; Jiang et al., 2017; Schiwon et al., 2018; Martinez et al., 2022; Zhang et al., 2023). In this assay, genomic DNA is isolated from the treated cells following CRISPR-mediated gene editing. The indel mutagenesis is determined based on the DNA fragments detected after the CRISPR/Cas9-mediated cleavage of the HBV DNA following T7E1 digestion of the specific PCR products. The indel mutations can also be detected by other techniques, such as the Indel Detection Amplicon Analysis (IDAA) and the Tracking of Indels by Decomposition (TIDE) assay, which is more sensitive and yields accurate results (Sentmanat et al., 2018). The mutation rate is calculated based on the grayscale intensity of the DNA band as follows: % gene modification = 100 × (1-(1-fraction cleaved)^1/2^), the fraction cleaved refers to the percent of nuclease-specific cleavage products. In addition, the PCR products can be cloned into a TA vector or directly sequenced by high-throughput DNA sequencing and analyzed by *in silico* bioinformatics tools (O'Geen et al., 2015; Morgens et al., 2017).

### On-target DNA sequencing

On-target DNA sequencing is a direct way to verify specific CRISPR-mediated gene-editing events. For this purpose, on-target amplicons are usually amplified via conventional PCR with a proofreading DNA polymerase and primers designed to flank target sites of gRNA/Cas9 for Sanger DNA sequencing and/or next-generation sequencing (NGS). Data analysis can be performed via BioEdit (Hall, 1999). In this case, contigs can be assembled using the CAP contig assemble program, and on-target amplicons are aligned with ClustalW multiple alignment. Notably, reports from CRISPR/Cas9-mediated HBV gene editing revealed that single nucleotide insertions and deletions dominated all other mutations in cccDNA following Cas9 cleavage (Seeger and Sohn, 2014; Seeger and Sohn, 2016) and that deletion was more common than insertion, which may result in the deletion of 1–10 nucleotides (Seeger and Sohn, 2014; Kennedy et al., 2015; Seeger and Sohn, 2016; Li H. et al., 2017; Scott et al., 2017). PAM sequences can also be removed after DNA repair by the NHEJ pathway (Seeger and Sohn, 2014; Kennedy et al., 2015; Seeger and Sohn, 2016).

The efficiency and specificity of HBV gene editing by CRISPR/Cas9 at the designated sites within the HBV sequences can be verified by NGS with single-cell DNA analysis. The excision efficiency in the HBV cccDNA segments is calculated as a ratio of the number of the sequence-verified fragments to all fragment numbers for each denoted experimental condition. With this definition, the excision efficiency is considered as frequentist probability, i.e., the ratio of the frequency of occurrence of the event of interest to the total number of experimental repeats. This interpretation of excision efficiency provides a predictive value, as it can be used to set a prior expectation on the success rate of each treatment for the excision of the desired segments of HBV cccDNA, and thus indicates the likelihood of successful drug treatment. Hierarchical clustering can be performed on the efficiency values of truncation events under different treatments and a scheme may be generated to group the efficiency values into a multilevel cluster tree represented by a dendrogram. Because the disruption and mutagenesis in the HBV genome (cccDNA) mediated by the CRISPR/Cas9 contribute to the antiviral effects, a heat map based on the analysis will offer a predictive value for HBV elimination. It should be mentioned that the T7E1 assay and Sanger DNA sequencing methods are essential for verifying gene editing at target sites and checking for off-target effects; however, these approaches can be somewhat biased because of one’s knowledge of potential sites of interest. On the other hand, approaches using NGS of the whole genome are unbiased methods and more sensitive, allowing for greater detection of the CRISPR/Cas9-mediated gene editing, both on and off targets (O'Geen et al., 2015; Morgens et al., 2017).

### Silico-protein prediction

The edited nucleotide sequences of the on-target genes can be in silico-translated to amino acid sequences using the Expasy Translate tool (web.expasy.org/translate/) for peptide structure prediction of the target genes’ encoded proteins (Gasteiger et al., 2003). Notably, in addition to frameshift mutations following CRISPR/Cas9-mediated gene editing and DNA damage repair by the NHEJ pathways, the resulting amino acid sequence downstream of the edited site can be substantially changed and even generate a shorter protein if a stop codon is introduced. The predicted amino acid sequences can be aligned using the Bioedit program (Hall, 1999) and the secondary structure can be modeled along with the 3D structure using SWISS-MODEL (swissmodel.expasy.org/interactive), and their binding functions can be predicted using the ProNA2020 code (Qiu et al., 2020) from PredictProtein (predictprotein.org) to observe their binding ability (Yachdav et al., 2014).

## Means used to examine off-target effects of CRISPR-mediated HBV gene editing

The CRISPR/Cas9 system can lead to off-target effects, which pose a risk to genome integrity (Morgens et al., 2017; Fu et al., 2013; Pattanayak et al., 2013). Off-target activity is defined as nonspecific cleavage at DNA sites outside of the target sequences, occurring in both the viral and human genomes. This nonspecific cleavage can result in unintended mutations, increasing the risk of genome instability and cellular carcinogenesis.

### Bioinformatic tools for off-target analysis

Only a few studies have examined the off-target effects of HBV-specific gRNAs by either genomic cleavage detection assay or deep sequencing, which is more sensitive in identifying potential off-target sites (Ramanan et al., 2015; Karimova et al., 2015; Li H. et al., 2017; Martinez et al., 2022). To ensure safety, gRNAs must be designed to specifically target HBV DNA with minimum or no off-targets within the human genome, which can be verified by web-based applications such as CRISPOR or E-CRISP (Concordet and Haeussler, 2018). Using CRISPOR (http://crispor.tefor.net) or E-CRISP (http://www.e-crisp.org) design, the specificity score (measures the uniqueness of a gRNA in the genome, ranging from 0–100 with 100 being the best (Hsu et al., 2013)) and anticipated efficacy score (provides how well the target gene is edited, ranging from 0–100 with 100 being the best (Doench et al., 2016)) must be determined first, so potential off-targets in both HBV and human genomes can be examined (Liu et al., 2018). Bioinformatic tools like CRISPRoff, CRISPRmit, and Cas-OFFinder can also predict potential off-target sites based on sequence similarity to the gRNA target sequence. Genome-wide silico prediction using computational analysis across the entire HBV and human genomes can be used to identify sequences that closely match the gRNA, allowing researchers to prioritize potential off-target sites for further analysis. Off-targets in the human genome can be further analyzed with Verify Guide Design from SYNTHEGO (https://design.synthego.com/#/validate) and CRISPR Off-Target Effects Analysis (https://www.creative-biogene.com/crispr-cas9/).

To ensure a thorough assessment of potential off-target effects, more than one hundred predicted sites that may be prone to DNA cleavage should be analyzed (Cameron et al., 2017; Koo et al., 2015). The objective is to identify both on-target and any off-target sites that might be induced by CRISPR/Cas9. This can be carried out by identifying genomic alterations, including structural variants (SVs), single nucleotide polymorphisms (SNPs), copy number variants (CNVs), and indel mutations under different treatments, and then comparing them to all potential off-targets. After thorough quality control steps, the resulting paired-end short-reads can be mapped to the human reference genome (Human_G1K-V37) by utilizing the Burrows-Wheeler Aligner (BWA) algorithm. Subsequently, the ratio of on-target and off-target effects can then be calculated.

### Cellular assays for cytotoxic effects

At the cellular level, hepatocyte cell lines and PHHs are transfected with CRISPR/Cas9 targeting HBV, drug cytotoxicity can be examined by various molecular assays, such as the CellROX assay (for oxidative stress in cells), mitoSOX (for mitochondrial ROS production), MTT proliferation assay (for cell viability and proliferation), LDH release assay (for cellular cytotoxicity), and Av/7AAD assay (for cell apoptosis). Using these methods, we have shown that the synthetic gRNA/Cas9 system can specifically and efficiently disrupt HBV genomes without causing apparent cytotoxicity (Zhang et al., 2023). Therefore, after on-target efficacy assessment and before using the selected gRNAs in humans, it is necessary to thoroughly examine whether any identified cytotoxic effects may be occurring independently of CRISPR/Cas9 activity and whether they are likely to lead to negative consequences.

## Future directions and challenges of CRISPR-based HBV gene therapy

Current HBV therapies rarely eliminate chronic HBV infection, primarily due to the persistence of cccDNA, which serves as the HBV replication template, exhibits extraordinary stability within infected hepatocytes, and is refractory to the current treatments. Given the significant morbidity and mortality associated with chronic hepatitis B, including complications such as liver cirrhosis and hepatocellular carcinoma, the pursuit of curative HBV therapy remains paramount. Although clinical data about the efficacy of CRISPR/Cas9 in humans are lacking, considering that the system depends only on the delivery of gRNA and Cas9 into target cells with an endogenous NHEJ pathway, we believe that the studies showing profound suppression of HBV replication using the CRISPR/Cas9 technology are promising to be clinically tested in patients with chronic hepatitis B. However, ongoing investigations into CRISPR-based HBV therapy encounter various limitations and hurdles. These encompass challenges related to delivery efficiency, off-target effects, immune responses, and the persistence of cccDNA. Addressing these obstacles is imperative to advancing CRISPR-based HBV gene therapy toward clinical application and achieving the ultimate goal of curing chronic HBV infection.

### Future directions and challenges

Future research should take into consideration the following challenges in designing CRISPR/Cas9-mediated HBV gene therapy:

I) Identifying optimal target sites: Selecting HBV cccDNA target sites critical for viral replication and gRNA/Cas9 accessibility is essential for the success of CRISPR-mediated HBV gene disruption and inactivation. Extensive profiling of potential Cas9 target sites on HBV cccDNA can uncover optimal target sites based on cccDNA accessibility and gRNA binding properties.
II) Developing authentic HBV models: HBV cell and animal models to be employed for evaluating the antiviral effects of the CRISPR/Cas9 system should produce authentic HBV cccDNA in HBV-infected hepatocytes *in vivo.* Therefore, more versatile and affordable HBV evaluation models that can mimic clinically relevant scenarios need to be further developed.
III) Improving cccDNA quantification: While Southern blot remains the “gold standard” method for measuring cccDNA, it is neither sensitive nor quantitative. Due to the abundance of rcDNA in HBV-infected hepatocytes, the Hirt DNA extraction and DNase digestion to remove rcDNA plus PCR-based cccDNA assay using “cccDNA-specific primers” may not measure cccDNA levels accurately. Therefore, developing reliable cccDNA quantification methods is necessary to evaluate CRISPR/Cas9-mediated anti-HBV efficacy accurately.
IV) Enhancing CRISPR/Cas9 specificity and safety: Off-target editing of the human genome by CRISPR/Cas9 raises safety concerns in humans (Fu et al., 2013; Pattanayak et al., 2013). Designing efficient gRNAs requires the selection of specific target sequences with low off-target activity. While SpCas9 remains the most used and studied Cas9, SpCas9 is known to have the largest potential for off-target activity compared with other Cas9 orthologs. Thus, orthologs with improved specificities (such as StCas9) have been recommended for testing in clinical trials, considering safety concerns (Liu et al., 2018; Kostyusheva et al., 2019).
  Alternatively, the issue of off-target editing may be solved by using a D10A mutated “nickase” variant of the Cas9 enzyme, which generates only a single-strand DNA break (nick), combined with paired gRNAs targeting the opposite strand of the DNA double helix to generate targeted DNA double-stranded breaks with high specificity (Cho et al., 2014; Ran et al., 2013; Mali et al., 2013). For this reason, the double-nicking approach can improve target specificity by up to 1,500-fold compared with the Cas9 wild-type protein (Cho et al., 2014; Ran et al., 2013; Mali et al., 2013). This significant improvement of Cas9 enzyme fidelity is of great interest concerning future therapeutic application of Cas9 nuclease in humans. While the activity of CRISPR/Cas9 nickase-mediated disruption and inactivation of HBV has been tested (Karimova et al., 2015; Sakuma et al., 2016; Suzuki et al., 2021), it remains to be seen whether the Cas9 nickase with paired gRNAs can eliminate intrahepatic cccDNA efficiently and whether it can be used for the curative treatment of CHB patients. The engineered Cas nucleases, including base editor and prime editor, are also under active investigation for their potential for being applied to therapeutics.
  Cleavage of integrated HBV genomes by CRISPR/Cas9 also results in DSBs in the host genome, which may cause large deletions and chromosomal rearrangements, leading to serious concern because this can cause instability of host genome, loss of heterozygosity, and carcinogenesis (Seeger and Sohn, 2014; Ramanan et al., 2015; Karimova et al., 2015; Li et al., 2016). In pioneering studies by Karimova et al. (Karimova et al., 2015) and Li et al. (Li H. et al., 2017), integrated HBV DNA was disrupted using an integrated HBV reporter sequence in Hela and HEK293 cell lines and a stable HBV cell line (HepG2.A64).
  Recently, a novel CRISPR/Cas9-derived base-editing (BE) strategy has been used to generate precise C-T/G-A conversion, in which the dCAs9-deaminase construct was fused with an uracil glycosylase inhibitor (UGI) that suppresses uracil excision following deamination, to prevent the reversion of the U:G pair to a C:G pair. Since then, a growing number of modified BE strategies have been developed to improve various aspects of BE tools (Schatoff et al., 2019). Theoretically, BE targeting nucleotides without DSBs of DNA should reduce the risk of genome rearrangement and carcinogenesis, however, Cas9-mediated BE can affect the stability of the host genome and thus should be carefully evaluated. Yang et al. (2020) demonstrated that CRISPR/Cas9-mediated BE is a potential strategy to cure HBV infection via permanent inactivation of the integrated HBV DNA and cccDNA through inducing nonsense mutations and premature stop codons of HBV genes without generating DSBs in the host genome. Nevertheless, the off-target effects of the CRISPR/Cas9-mediated HBV gene editing events need to be examined stringently using genome-wide evaluation approaches before its clinical application (Morgens et al., 2017; Fu et al., 2013; Pattanayak et al., 2013).
V) Improving CRISPR/Cas9 delivery systems: Delivery of gRNA/Cas9 modalities using clinically relevant viral vectors (e.g., liver tropic AAVs) requires additional modifications such as switching to smaller Cas9 orthologs to save packaging size or using HCAdVs. Using non-viral delivery methods (e.g., LNPs or engineered exosomes) to deliver gRNA/Cas9 RNPs is under active development and evaluation. Therefore, improving the specificity and efficacy of CRISPR/Cas9 delivery to liver hepatocytes is desperately needed to move this field forward.
  For safety considerations, a transient system using synthetic gRNA/Cas9 RNPs has the benefits of inducing faster cleavage with minimum off-target activity and immunogenicity; however, delivery of these therapeutic RNPs remains a challenge. Ideally, the CRISPR/Cas9 modalities should be delivered to all HBV-infected hepatocytes to achieve a complete cure. However, the large CRISPR/Cas9 complex is charged and cannot readily be transported to HBV-infected hepatocytes. The currently available viral and non-viral CRISPR/Cas9 delivery systems are far from ideal for clinical applications. Addressing these challenges will be pivotal in advancing CRISPR-based HBV gene therapy toward clinical application and achieving the goal of curing chronic HBV infection.

### Considerations for CRISPR-based HBV gene therapy

The following treatment strategies should be considered when designing CRISPR-based gene therapies for HBV cure:

A) Multiplexing strategy. Previous studies suggested that a sgRNA for Cas9 cleavage might be insufficient to completely inactivate HBV cccDNA (Seeger and Sohn, 2014; Seeger and Sohn, 2016). Theoretically, even a single copy of replication-competent cccDNA could lead to viremia rebound after stopping the antiviral therapy. Therefore, a complete (virologic) cure aims to eliminate all HBV cccDNA as well as integrated HBV DNA from every infected hepatocyte. According to the current reported data, utilizing the CRISPR/Cas9 technology could suppress but not eliminate all the persistent HBV genomes from infected hepatocytes. Thus, a combination of gRNAs targeting multiple loci on the HBV genomes is imperative to achieve HBV eradication. Several investigators reported that a multiplexing approach could generate multiple indel mutations and intensity antiviral effects (Lin et al., 2014; Dong et al., 2015; Liu et al., 2015; Ramanan et al., 2015; Zhang et al., 2023), suggesting that this strategy may further maximize the CRISPR-mediated HBV suppression effects. Multilocus targeting not only induces multiple site mutations but can also cause large deletions between target loci, and thus increase the chance of viral genomic disruption (Sakuma et al., 2016). The multiplexing strategy was also effective for HBV genomes of different genotypes from different geographic regions (Lin et al., 2014). We (Zhang et al., 2023) and others (Martinez et al., 2022) reported that CRISPR/Cas9 could significantly reduce the levels of HBV products and meanwhile generate transcriptionally active episomal variants. Therefore, simultaneous cleavage of multiple sites can be achieved by using multiplex gRNAs that increase the efficiency of CRISPR/Cas9 in disrupting HBV genomes and reducing the emergence of viral mutations escaping cleavage.
B) Functional HBV cure. Functional curing of HBV can be achieved by eliminating the majority of HBV DNA and HBsAg products despite the presence of a trace level of intrahepatic cccDNA, which may be eventually cleared by host immunity – and lead to a state of immunological cure or clinical cure. Notably, a low level of cccDNA was detected in the liver of patients who had spontaneously resolved acute HBV infection decades prior (Sells et al., 1987; Xu et al., 2021; Gripon et al., 2002; Ladner et al., 1997; Yan et al., 2012), suggesting that while complete elimination of the intrahepatic cccDNA may remain an unreachable goal, immunological control and clinical resolution of HBV infection is feasible. Thus, a functional or clinical HBV cure with undetectable or substantially reduced cccDNA levels is now deemed a key outcome.
C) Combined treatment for HBV cure. If combined with NAs that effectively suppress HBV replication, the CRISPR/Cas9 system may have the potential to eradicate the persistent HBV cccDNA in CHB patients, raising the hope for curing chronic HBV infection. Indeed, Kayesh et al. (Kayesh et al., 2020) have shown that AAV-delivered CRISPR/Cas9 could enhance the entecavir-mediated antiviral effects, suggesting that while these two approaches follow different modes of action, they can be used in combination for HBV curative treatment. A combination of effective antiviral drugs (e.g., NAs) and/or adjuvant immunotherapy (e.g., IFN-α) appears paramount to meeting this overarching goal of functional or clinical HBV cure. Therefore, achieving efficient delivery of gene editing therapeutic modalities, ensuring their specificity to disrupt HBV target genes, minimizing off-target effects, and integrating gene editing modalities with antiviral and immunologic drugs may ultimately lead to curing chronic HBV infection.

### Concluding remarks

The development of gene editing technologies against HBV has advanced to the breakthrough stage over the past decade, offering unprecedented possibilities for novel combination treatments and eradication of this chronic infectious disease. In this era of increasingly personalized medicine, the prospects of designing HBV gene editors tailored to individual patient circumstances appear imminent. However, alongside the robust development and refinement of these new technologies come new challenges and questions. Further studies are imperative to address these challenges and ensure that HBV gene editing is undertaken with caution to avoid unforeseen consequences. It is crucial to ensure that the pursuit of a cure leads to an intentional, rather than an accidental, outcome. Continued research and careful consideration will be essential as we navigate the complex landscape of CRISPR-based HBV gene editing in pursuit of a lasting solution to this global health challenge.

## Summary

**FIGURE 5:**
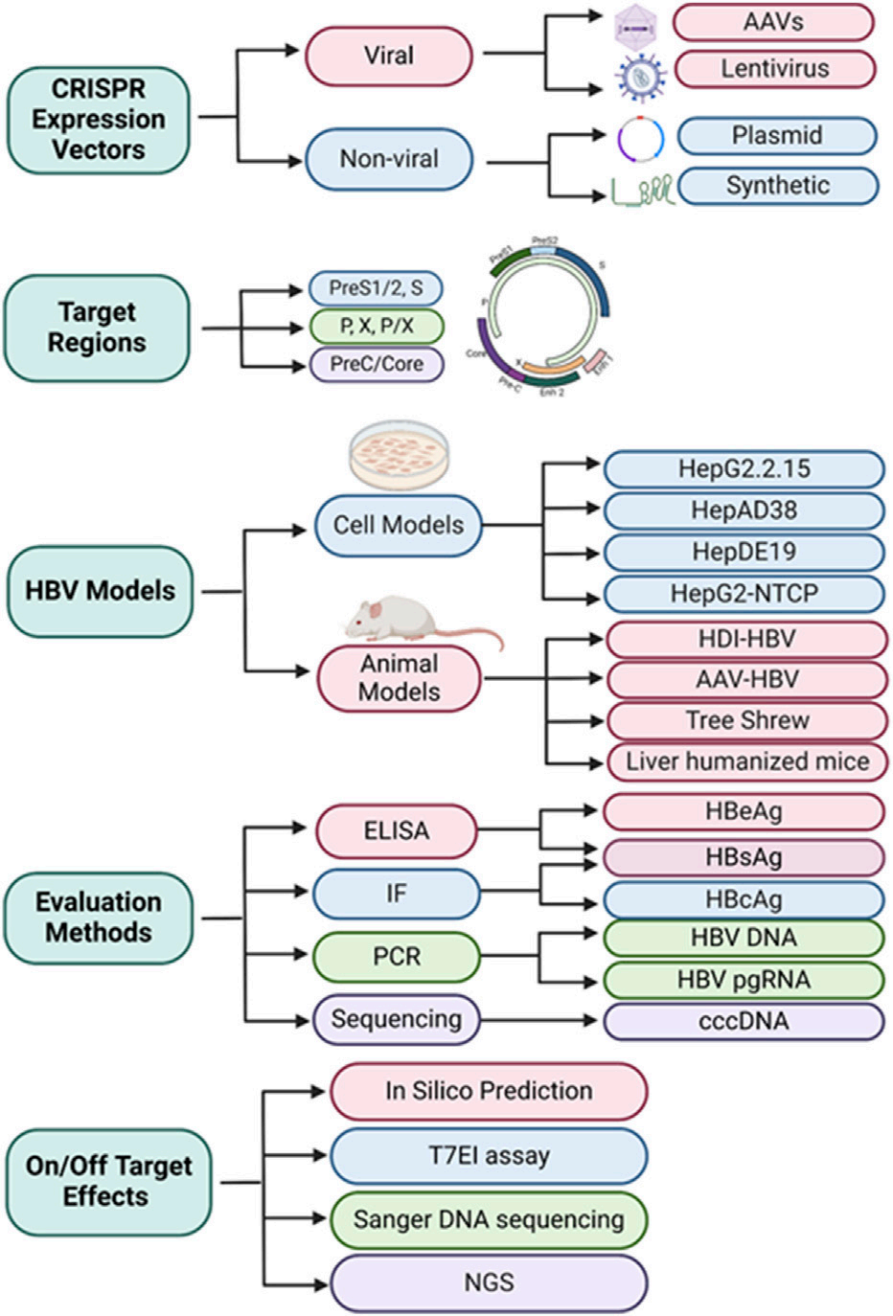
Algorithm of CRISPR/Cas9-mediated HBV gene disruption and inactivation.

This article reviewed the CRISPR/Cas9 technology to disrupt and inactivate HBV genome. We summarized the tools employed in designing guide RNAs (gRNAs) targeting HBV genomes, the vehicles used for expressing and delivering CRISPR/Cas9 components, the models used for evaluating CRISPR-mediated HBV gene disruption, the methods used for assessing antiviral and off-target effects induced by CRISPR/Cas9 (Figure 5), and the prospects of future directions and challenges in leveraging this HBV gene-editing approach to advance the HBV treatment toward a clinical cure.

## Glossary

7-AAD: 7-Aminoactinomycin D
AAV: Adeno-associated virus
AuNPs: gold nanoparticles
Av: annexin V
BE: base-editing
BWA: Burrows-Wheeler Aligner
cccDNA: covalently closed circular DNA
CHB: chronic hepatitis B
CNVs: copy number variants
CRISPR: Clustered Regularly Interspaced Short Palindromic Repeats
crRNA: CRISPR RNA
ddPCR: droplet digital polymerase chain reaction
DHBV: duck hepatitis B virus
DR1 or DR2: direct repeat 1 or direct repeat 2
DSBs: double-stranded breaks
Enh1 or Enh2: enhancer 1 or enhancer 2
ETSU: East Tennessee State University
FAH: Fumaryl acetoacetate hydrolase
FnCas9: Francisella novicida-derived Cas9
GFP: green fluorescence protein
gRNA: guide RNA
HBcAg: hepatitis B core antigen
HBeAg: hepatitis B e antigen
HBsAg: hepatitis B surface antigen
HBV: hepatitis B virus
HBxAg: hepatitis B x antigen
HCAdVs: high-capacity adenoviral vectors
HCC: hepatocellular carcinoma
HDI: hydrodynamic injection
HDR: homology-directed repair
HMCs: hematopoietic stem cells
HPCs: hepatic progenitor cells
IDAA: Indel Detection Amplicon Analysis
IFN-α: interferon-alpha
IF: immunofluorescence
Indel: insertion/deletion
LNPs: lipid-like nanoparticles
LDH: lactate dehydrogenase
NAs: nucleos(t)ide analogues
NGS: next-generation sequencing
NHEJ: non-homologous end joining
NmCas9: *Neisseria* meningitidis-derived Cas9
NRG: NOD.Rag1KO.IL2Rγc
NTCP: sodium taurocholate co-transporting polypeptide
ORF: open reading frame
PAM: protospacer adjacent motifs
PARP: Poly (ADP-ribose) polymerase
PEG-IFN: pegylated interferon
PEI: polyethylenimine
pgRNA: pre-genomic RNA
PHH: primary human hepatocytes
P/Pol: polymerase
rcDNA: relaxed circular DNA
RFP: red fluorescence protein
RNP: ribonucleoprotein
RT: reverse transcriptase
SaCas9: *Staphylococcus* aureus-derived Cas9
sgRNA: single gRNA
SNPs: single nucleotide polymorphisms
SpCas9: *Streptococcus* pyogenes-derived Cas9
StCas9: *Staphylococcus* thermophilus-derived Cas9
T7E1: T7 endonuclease 1
TALENs: transcription activator-like effector nucleases
tet: tetracycline
Tg: transgenic
TIDE: Tracking of indels by decomposition
tracrRNA: trans-activating CRISPR RNA
UGI: uracil glycosylase inhibitor
WHV: woodchuck hepatitis B virus
YMDD: tyrosine-methionine-aspartate-aspartate
ZFNs: zinc finger nucleases.
